# Crosstalk between SELENOF and the unfolded protein response in breast epithelium dictates cell fate

**DOI:** 10.1038/s41420-026-03285-7

**Published:** 2026-08-10

**Authors:** An Zhang, Abigail Rullo, Brenna Flowers, Olivia Sibu, Roudy C. Ekyalongo, Alan M. Diamond, Irida Kastrati

**Affiliations:** 1 Dept. of Cancer Biology, Loyola University Chicago Stritch School of Medicine, Maywood, IL USA; 2 Dept. of Pathology, University of Illinois Chicago, Chicago, IL USA

**Keywords:** Senescence, Breast cancer

## Abstract

SELENOF is an understudied selenoprotein that contains selenium in the form of selenocysteine. Our previous findings indicate that SELENOF likely functions as a tumor suppressor in breast cancer; however, its precise molecular role and regulatory mechanisms remain poorly understood. SELENOF localizes to the endoplasmic reticulum (ER) through a strong association with UDP-glucose glycoprotein glucosyltransferase 1 (UGGT1) and is thought to participate in protein folding quality control. Impaired protein folding efficiency leads to the accumulation of unfolded or misfolded proteins, thereby inducing ER stress and activating the unfolded protein response (UPR), an adaptive signaling network that restores ER homeostasis. Whether SELENOF modulates the UPR specifically in mammary epithelial cells and how this impacts cellular outcomes remain unknown. Here, we demonstrate that SELENOF establishes a feedforward regulatory loop with the inositol-requiring enzyme 1α (IRE1α) and X-box binding protein 1 (XBP1) arm of the UPR. SELENOF expression is upregulated by ER stress through the IRE1α-XBP1 pathway in normal immortalized human epithelial cells, murine mammary glands, and human breast cancer cells, thereby amplifying UPR signaling. In cancer cells, SELENOF overexpression exacerbates UPR activation, leading to IRE1α-dependent cell death. Conversely, SELENOF loss in normal breast epithelial cells limits the UPR to adaptive levels, allowing cells to evade senescence. Moreover, silencing UGGT1, a key enzyme in the glycan quality control pathway and interacting partner of SELENOF, reduced SELENOF protein levels and phenocopied its effects in limiting stress-induced activation of the IRE1-XBP1s axis of the UPR. Collectively, these findings identify SELENOF as a critical modulator of the UPR and highlight a complex and context-dependent role for SELENOF and IRE1α-UPR in breast tumorigenesis.

## Introduction

Breast cancer remains the most frequently diagnosed malignancy and a leading cause of cancer-related deaths among women worldwide [1]. In the United States, more than 310,000 new cases are projected to be diagnosed this year, leading to over 40,000 deaths [2]. Despite major advances in clinical management, including endocrine therapy, targeted agents, and chemotherapy, a substantial proportion of patients ultimately develop resistance and die from the disease. Selenium status was shown to strongly correlate with survival among breast cancer patients [3], indicating that certain selenoproteins, including SELENOF, may play a functional role in tumor progression. Notably, SELENOF mRNA expression is markedly reduced in late-stage breast tumors compared to normal breast tissue, and lower SELENOF levels are associated with poor patient outcomes [4]. Experimental studies further show that SELENOF overexpression suppresses breast cancer cell growth and promotes cell death, specifically via apoptosis and autophagy, whereas its loss enhances proliferation and confers resistance to apoptosis and autophagy in non-tumorigenic breast cells [4, 5].

Despite continued investigation, the precise function of SELENOF remains elusive. Mice lacking Selenof expression develop early-onset cataracts [6], and their B cells secrete elevated levels of nonfunctional IgM [7]. These observations led to the hypothesis that SELENOF facilitates the folding and maturation of secretory proteins, particularly those rich in disulfide bonds. This function is likely conferred by its selenocysteine residue; compared to cysteine, the lower *p*Ka of selenocysteine within SELENOF’s thioredoxin-like domain and its predicted redox potential suggest reductase or protein disulfide isomerase activity [8]. SELENOF typically resides in the endoplasmic reticulum (ER) lumen [9] by forming a high affinity 1:1 complex with UDP-glucose glycoprotein glucosyltransferase 1 UGGT1 [10], the folding sensor of the calnexin cycle, and enhances UGGT1’s enzymatic activity in glycoprotein maturation and redox quality control [11, 12]. Together, these findings suggest a broader role for SELENOF in modulating the ER stress response. However, whether SELENOF contributes to protein quality control and ER stress regulation in the mammary gland or in breast cancer cells remains unknown.

The ER is a central organelle that governs essential cellular processes, including protein synthesis, folding, and post-translational modification, as well as lipid metabolism and calcium homeostasis [13]. When protein homeostasis is disrupted, ER-resident transmembrane sensors—inositol-requiring enzyme 1 alpha (IRE1α), protein kinase R (PKR)-like endoplasmic reticulum kinase (PERK), and activating transcription factor 6 (ATF6)—activate downstream signaling pathways that either restore proteostasis to support cell survival or trigger apoptosis under prolonged stress [14]. Collectively, these signaling cascades alleviate ER stress by transiently reducing protein synthesis and inducing transcriptional programs that enhance the ER’s folding capacity, a process known as the unfolded protein response (UPR).

IRE1α represents the most evolutionarily conserved branch of the UPR. Substantial evidence supports a pro-tumorigenic role for the UPR transducer IRE1α and its downstream effector, X-box binding protein 1 (XBP1). The IRE1α-XBP1 axis has been shown to promote tumor progression in both hormone-dependent (HR + ) and triple-negative breast cancers (TNBC). In HR+ breast cancer cells, XBP1 enhances growth and proliferation by activating estrogen receptor signaling [15]. In TNBC, IRE1α displays constitutively high basal activity, resulting in persistent XBP1 activation, which dimerizes with hypoxia-inducible factor 1 alpha (HIF1α) to drive the expression of hypoxia-responsive genes that promote tumor growth and angiogenesis [16]. Moreover, UPR activation has been linked to resistance against diverse therapies, including radiation, tamoxifen, and conventional chemotherapeutic agents [17]. Conversely, UPR activation can sensitize breast cancer cells to apoptosis in response to certain targeted therapies, such as bortezomib or combined lapatinib and obatoclax treatment [18, 19]. These seemingly opposing outcomes underscore the context-dependent nature of UPR signaling and highlight the need to delineate the specific contributions of each UPR arm and their regulators to breast cancer pathophysiology.

## Results

### Loss of SELENOF does not change baseline UPR, whereas overexpression of SELENOF elevates UPR to inhibit breast cancer cells in an IRE1α-dependent manner

**Fig. 1:**
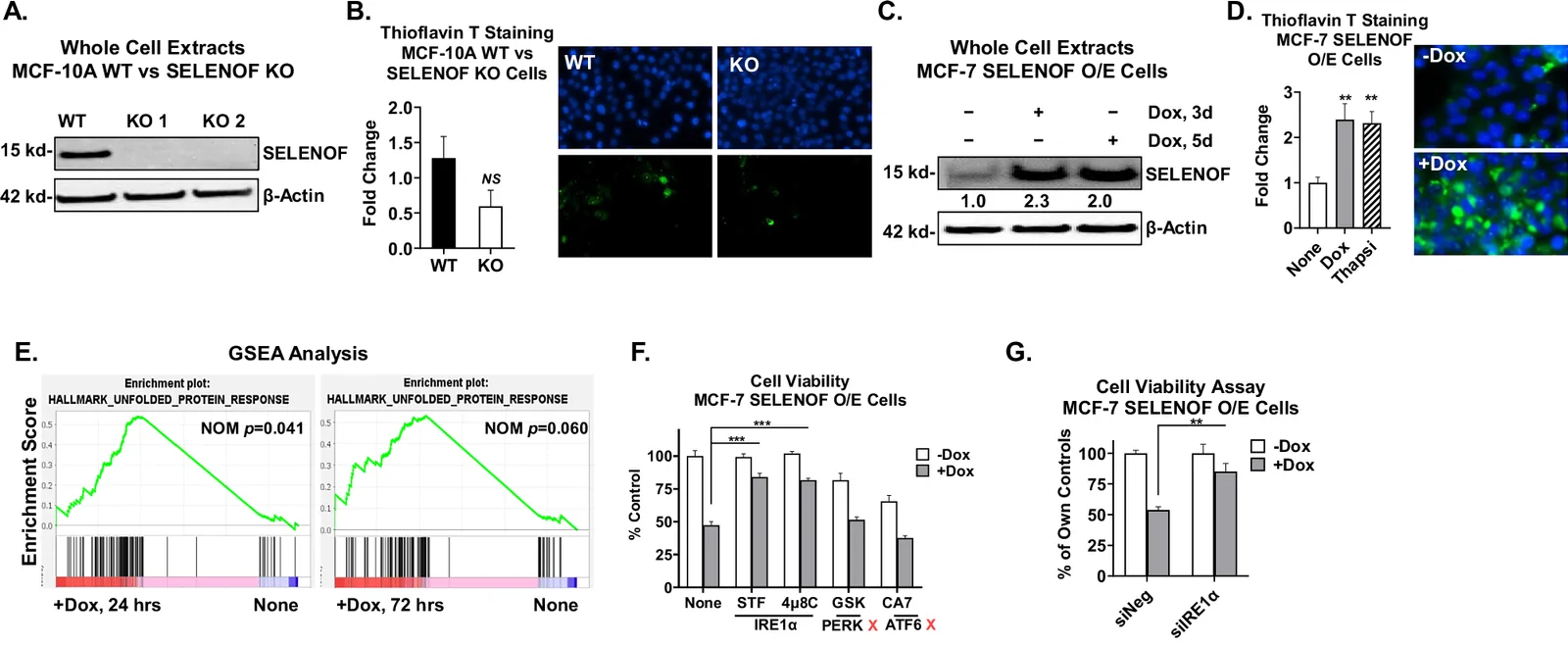
Loss of SELENOF does not disrupt intracellular proteostasis, however its overexpression increases proteotoxic burden leading to cell death via IRE1α signaling. **A** SELENOF levels in immortalized normal epithelial cells MCF-10A wild type (WT) and in CRISPR/Cas9 SELENOF knockout cells (two clones; KO 1, KO 2) were determined by western blotting. β-Actin is a loading control. **B** Quantification and representative images of basal misfolded proteins by thioflavin T staining in MCF-10A WT and SELENOF KO cells are shown. **C** SELENOF levels in MCF-7 cells stably transduced with a Tet-On SELENOF overexpression (O/E) system were determined by western blotting. Protein densitometry values are shown below the bands. Cells were treated with doxycycline (Dox, 1 μg/mL) for 3 or 5 days. **D** Quantification of misfolded proteins in MCF-7 SELENOF cells by thioflavin T staining. Cells were treated with 1 μg/mL doxycycline for 4 days. ER stress inducer thapsigargin (Thapsi, 1 μM for 24 h) was used as a positive control. **E** RNA sequencing and gene set enrichment analysis conducted on RNA isolated from MCF-7 SELENOF cells treated with 1 μg/mL doxycycline for 24 or 72 h indicate enrichment for the UPR gene signature. **F** Cell viability of MCF-7 SELENOF cells was determined by crystal violet staining. Cells were treated with or without 1 μg/mL doxycycline and IRE1α inhibitor (STF-083010, 2 μM; 4μ8C, 0.1 μM),), PERK inhibitor (GSK2606414, 2 µM) or ATF6 inhibitor (CEAPIN-A7, 6 µM) for 3 days. Data was normalized to the none control set as 100%. **G** Cell viability was evaluated in cells transfected with siNeg or siIRE1α, 20 nM each, and treated with 1 μg/mL doxycycline for 3 days. Data was normalized to siNeg control set as 100%. Data represents the mean ± SEM of independent experiments (*n* = 3). **P ≤ 0.01, ***P ≤ 0.001. NS is not significant.

To investigate the role of SELENOF, we employed multiple complementary models: (i) deletion of SELENOF in MCF-10A normal breast epithelial cells, which express high endogenous levels of SELENOF; (ii) overexpression of SELENOF in MCF-7 breast cancer cells, which have low endogenous expression; and (iii) mammary glands isolated from wild type and Selenof knockout mice. Given the proposed role of SELENOF in protein folding and redox quality control, we hypothesized that loss of SELENOF would increase proteotoxic burden and trigger UPR in breast epithelial cells. To test this, we used control MCF-10A cells with wild-type (WT) SELENOF expression and two independent SELENOF knockout (KO) clones (KO 1 and KO 2) generated by CRISPR/Cas9 editing, as previously described [5] (Fig. 1A). The extent of protein misfolding was assessed using thioflavin T staining and fluorescence microscopy. Thioflavin T is a fluorescent dye that binds β-sheet–rich protein aggregates, producing a characteristic increase in fluorescence intensity. Using this assay, we found that loss of SELENOF had no significant effect on the basal accumulation of misfolded proteins (Fig. 1B). In contrast, SELENOF overexpression in MCF-7 breast cancer cells using a Tet-ON inducible system reported before [4] produced the opposite effect (Fig. 1C) and led to significant misfolded protein levels comparable to those induced by thapsigargin, a known ER stressor (Fig. 1D). Of note, SELENOF expression in breast cancer cells is sensitive to selenium supplementation; treatment with sodium selenite increased SELENOF levels in a time-dependent manner, reaching levels comparable to those achieved by ectopic overexpression (Supplementary Fig. 1A). To assess the global transcriptomic impact of SELENOF overexpression, RNA sequencing and gene set enrichment analysis were conducted on RNA isolated from cells treated Dox for 24 h or 72 h and compared to untreated controls. Consistent with the accumulation of misfolded proteins (Fig. 1D), the UPR pathway was significantly enriched at both time points (Fig. 1E). As reported previously [4], SELENOF overexpression inhibits breast cancer cell proliferation and induces cell death (Fig. 1F, first two bars). To determine whether the UPR and its downstream signaling arms, IRE1α, PERK, and ATF6, mediate these effects, we employed pharmacological inhibitors targeting each pathway: STF083010 (STF) and 4μ8C for IRE1α, GSK2606414 (GSK) for PERK, and CEAPIN-A7 (CA7) for ATF6. The inhibitors’ effects on the respective arms of the UPR were confirmed following thapsigargin treatment; STF reduced XBP1s levels (Figs. 3D, 3 ^rd^ vs 4^th^ lanes), GSK reduced CHOP (Supplementary Fig. 1B, 2^nd^ vs 4^th^ lane), and CA7 attenuated BIP levels (Supplementary Fig. 1C, 2^nd^ vs 4^th^ lanes). Only IRE1α inhibition rescued the growth-inhibitory effects of SELENOF overexpression, whereas PERK or ATF6 inhibition had no effect (Fig. 1F). This finding was corroborated by genetic silencing of IRE1α using siRNA, which similarly reversed the SELENOF-induced growth inhibition in breast cancer cells (Fig. 1G, Supplementary Fig. 1D).

Notably, in MCF-10A cells where SELENOF deletion did not result in misfolded proteins (Fig. 1B), treatment with UPR inhibitors had no differential effect on cell viability based on SELENOF status (Supplementary Fig. 1E). Similarly, assessment of IRE1α signaling by western blotting and RT-QPCR showed no baseline changes in these cells (Supplementary Fig. 1F, G). Collectively, these results indicate that loss of SELENOF in normal breast epithelial cells did not activate UPR, whereas SELENOF overexpression increases proteotoxic stress and activates the UPR-IRE1α axis in breast cancer cells, and that IRE1α signaling is essential for meditating the inhibitory effects of SELENOF in this context.

### SELENOF facilitates activation of the IRE1α-XBP1s axis upon ER stress

**Fig. 2:**
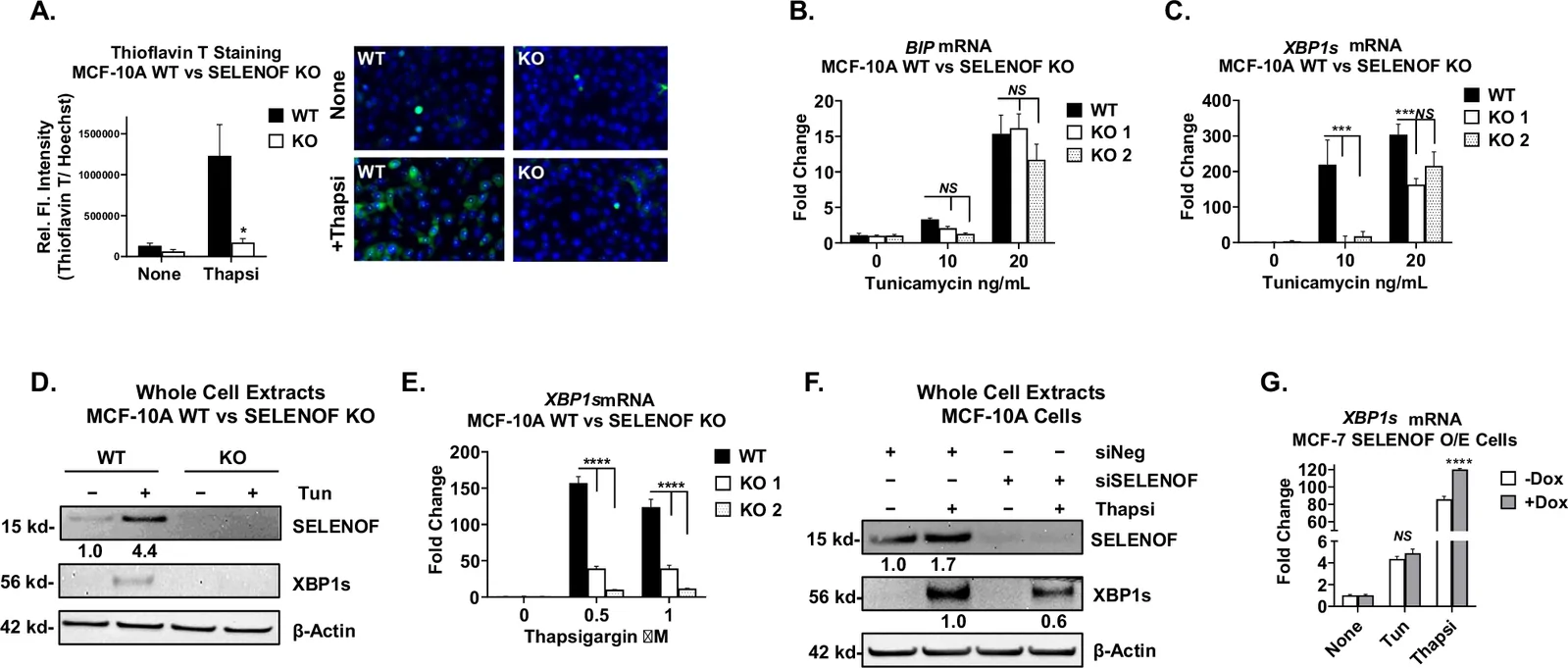
SELENOF is required for the full activation of IRE1α-XBP1s axis upon induction of ER stress. **A** Quantification of misfolded proteins by thioflavin T staining in MCF-10A WT and SELENOF KO cells. Cells were treated with or without 1 μM thapsigargin (Thapsi, 24 h) before staining. **B**, **C** *BIP* and *XBP1s* mRNA expressions were determined by RT-QPCR in MCF-10A WT and SELENOF KO cells treated with or without tunicamycin at indicated concentrations for 24 h. **D** SELENOF and XBP1s levels were determined in MCF-10A WT and SELENOF KO cells treated with or without 10 ng of tunicamycin for 6 h. Protein densitometry values are shown below the bands. **E** *XBP1s* mRNA expression was determined in MCF-10A WT and SELENOF KO cells treated with or without thapsigargin for 24 h. **F** SELENOF and XBP1s levels were determined in MCF-10A cells transfected with siNeg or siSELENOF, 10 nM each for 24 h, and then treated with or without 1 μM thapsigargin for 6 h. Protein densitometry values are shown below the bands. **G** *XBP1s* mRNA expression was determined in MCF-7 SELENOF cells treated with 1 μg/mL doxycycline (Dox) for 24 h, followed by tunicamycin (Tun, 10 ng) or thapsigargin (Thapsi, 1 μM) for another 24 h. Data represents the mean ± SEM of independent experiments (*n* = 3). *P ≤ 0.05, ***P ≤ 0.001, ****P ≤ 0.0001. NS is not significant.

Although loss of SELENOF did not alter the UPR under basal conditions in normal cells experiencing low ER stress, we investigated whether SELENOF modulates the cellular response under conditions of induced ER stress. To this end, we treated MCF-10A cells with thapsigargin, a sarco/ER Ca²⁺-ATPase (SERCA) inhibitor, or tunicamycin, an inhibitor of N-acetylglucosamine phosphotransferase. Thioflavin T staining revealed a significant accumulation of misfolded proteins following thapsigargin treatment in cells with intact SELENOF levels (WT) (Fig. 2A, black bars). In contrast, SELENOF KO cells exhibited markedly reduced misfolded protein accumulation under the same conditions (Fig. 2A, white bars), indicating that SELENOF is required for intracellular accumulation of misfolded proteins during ER stress. We next assessed the expression of BIP (also known as GRP78, glucose-regulated protein 78), a key ER chaperone that serves as an upstream sensor of the UPR by modulating activation of all three canonical branches, IRE1α, PERK, and ATF6. Tunicamycin treatment robustly upregulated *BIP* mRNA, whereas SELENOF deletion had little to no effect on this response (Fig. 2B).

To specifically evaluate IRE1α pathway activation, we measured spliced XBP1 (*XBP1s*) mRNA. Upon ER stress, activated IRE1α cleaves unspliced *XBP1* mRNA to generate XBP1s, a potent transcription factor that translocates to the nucleus to drive transcription of UPR target genes. Tunicamycin induced a concentration-dependent increase in *XBP1s* mRNA in WT cells (Fig. 2C, black bars), confirming IRE1α pathway activation under this condition. In contrast, SELENOF KO cells displayed a significantly impaired induction of *XBP1s* mRNA in response to tunicamycin treatment (Fig. 2C), and XBP1s protein accumulation was markedly reduced compared to WT controls (Fig. 2D, 2^nd^ vs. 4^th^ lanes). A similar effect was observed following thapsigargin exposure, where SELENOF KO cells exhibited significantly lower *XBP1s* mRNA expression compared with WT cells (Fig. 2E). This blunted response with loss of SELENOF extended to downstream XBP1s target genes, including *EDEM1* (ER degradation enhancing α-mannosidase like protein 1), which showed a parallel reduction in expression in SELENOF KO cells (Supplementary Fig. 2A).

Given the structural and functional homology between IRE1α and PERK, we also examined PERK, its downstream effector and transcription factor ATF4, as well as additional target genes, including CHOP and GADD34. Although thapsigargin-treated SELENOF KO cells showed a trend toward reduced *PERK*, *ATF4*, *CHOP*, and *GADD34* mRNA expression relative to wild-type (WT) cells (Supplementary Fig. 2B–E), these effects were less pronounced and less consistent than those observed for the IRE1α-XBP1s axis. As an alternative approach, SELENOF expression was silenced using siRNA, which similarly resulted in a significant reduction in XBP1s protein levels following thapsigargin treatment (Fig. 2F, 2^nd^ vs 4^th^ lanes). Conversely, overexpression of SELENOF in MCF-7 cells further enhanced thapsigargin-induced XBP1s mRNA expression (Fig. 2G). Collectively, these findings indicate that SELENOF is critical for mounting a robust UPR, with a particularly prominent role in facilitating activation of the IRE1α-XBP1s signaling axis during ER stress. Co-immunoprecipitation analysis demonstrated a direct interaction between SELENOF and BIP, consistent with their shared localization in the ER, but not with IRE1α (Supplementary Fig. 2F). These findings suggest that SELENOF modulates IRE1α-XBP1s signaling indirectly.

### SELENOF is upregulated by ER stress via IRE1α-XBP1s and amplifies UPR in vitro and in vivo

**Fig. 3:**
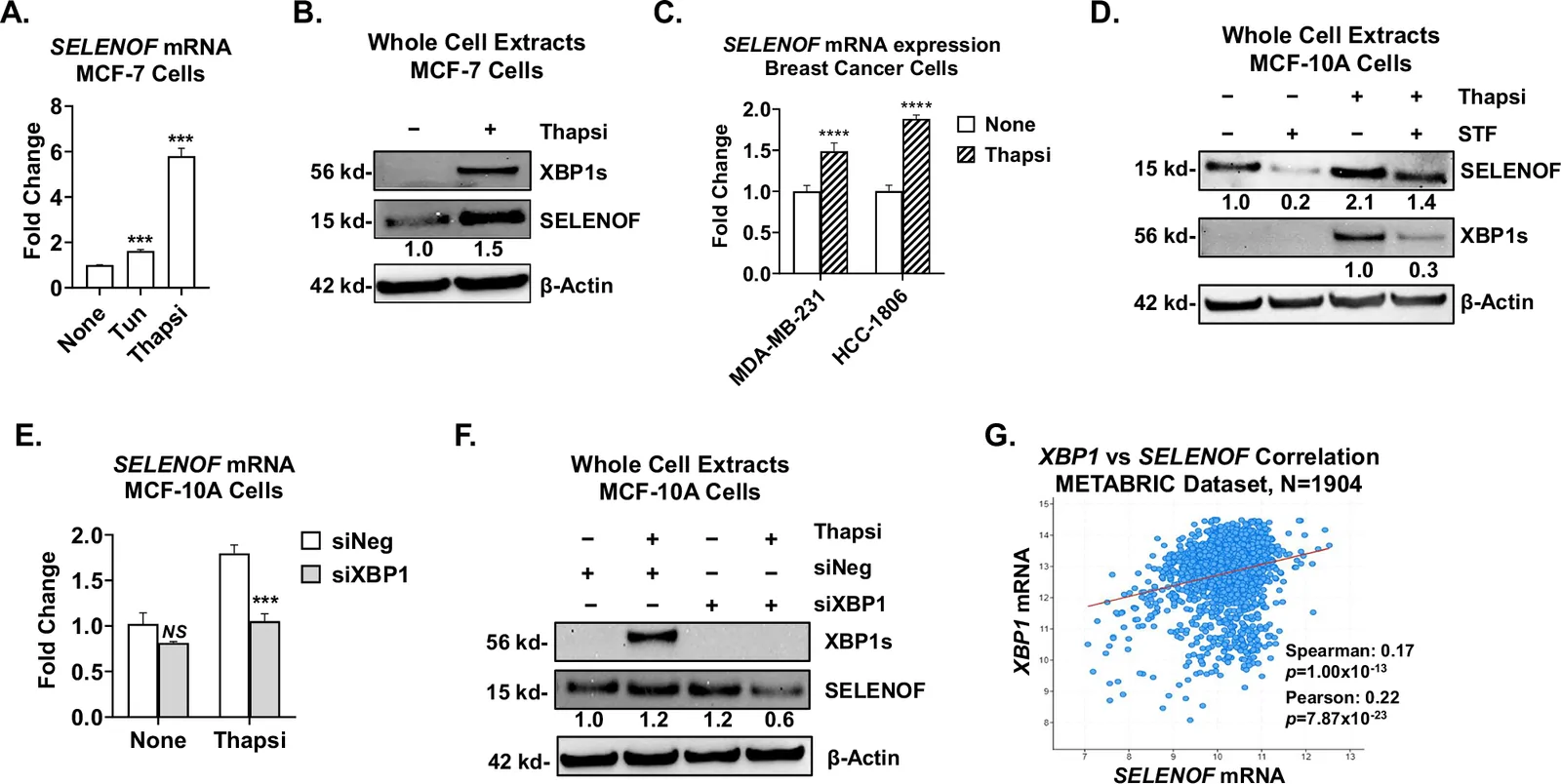
Activation of the IRE1α-XBP1s axis enhances SELENOF expression. **A** *SELENOF* mRNA expression was determined in MCF-7 cells treated with tunicamycin (Tun, 20 ng) or thapsigargin (Thapsi, 1 μM) for 24 h. **B** XBP1s and SELENOF levels in MCF-7 cells treated with or without 1 μM thapsigargin for 24 h. **C** *SELENOF* mRNA expression in breast cancer cell lines, MDA-MB-231 and HCC1806, treated with or without thapsigargin (Thapsi, 1 μM) for 24 h. **D** SELENOF and XBP1s levels were determined in MCF-10A cells. Cells were treated with or without 1 μM thapsigargin or the IRE1α inhibitor STF083010 (STF, 30 µM) for 6 h. Protein densitometry values are shown below the bands. **E** *SELENOF* mRNA expression was determined in MCF-10A cells transfected with siNeg or siXBP1 for 24 h followed by 1 μM thapsigargin treatment for another 24 h. **F** SELENOF and XBP1s levels in MCF-10A cells. Cells were first transfected with siNeg or siXBP1 followed by treatment with or without 1 μM thapsigargin for 6 h. Protein densitometry values are shown below the bands. **G** Correlation plot between *SELENOF* and *XBP1* mRNA expression from breast cancers patient samples within the METABRIC database, *n* = 1904 samples. Spearman and Pearson coefficients and *P*-values are indicated. Data represents the mean ± SEM of independent experiments (*n* = 3). ***P ≤ 0.001, ****P ≤ 0.0001. NS is not significant.

Beyond its known selenium responsiveness, little is understood about the regulation of SELENOF expression. We observed that ER stress inducers, including thapsigargin and tunicamycin, increased SELENOF protein levels in MCF-10A cells (Fig. 2D, F; 1^st^ vs 2^nd^ lanes) by 1.7-4.4-fold, prompting further investigation. In MCF-7 breast cancer cells, both compounds similarly upregulated *SELENOF* mRNA expression (Fig. 3A). This effect was mirrored at the protein level, where thapsigargin upregulated SELENOF in parallel with XBP1s activation (Fig. 3B). Comparable results were obtained in additional breast cancer cell lines, including MDA-MB-231 and HCC-1806, where thapsigargin treatment also upregulated SELENOF expression (Fig. 3C). Thapsigargin induced *XBP1s* mRNA serves as a positive control (Supplementary Fig. 3A).

Given SELENOF’s role in enhancing the IRE1α-XBP1s axis, we next investigated whether the IRE1α-XBP1s axis reciprocally regulates SELENOF in a feedforward manner. In MCF-10A cells, thapsigargin treatment led to increased SELENOF levels by 2.1-fold, whereas this induction was abolished by the IRE1α RNase inhibitor STF083010 (STF), concomitant with reduced XBP1s levels (Fig. 3D, 3 ^rd^ vs 4^th^ lanes). To directly test the role of XBP1, XBP1 expression was silenced by using siRNA. Under these conditions, thapsigargin-induced *SELENOF* mRNA (Fig. 3E) and protein (Fig. 3F) expression were both significantly attenuated. The reduction in *XBP1s* mRNA was confirmed as a positive control (Supplementary Fig. 3B).

To examine this regulatory relationship in human tumors, we analyzed the METABRIC breast cancer cohort (*n* = 1904). Although direct XBP1s expression data were unavailable, *XBP1* mRNA expression and those of two established XBP1s target genes, *EDEM1* and *DNAJB9* (DnaJ heat shock protein family B9), were examined. SELENOF mRNA positively correlated with all three, with the strongest associations observed with DNAJB9 and EDEM1 (Fig. 3G; Supplementary Fig. 3C, D), supporting a transcriptional link between XBP1s activity and SELENOF expression in breast cancer. Collectively, our data identify SELENOF as a downstream effector of ER stress that is transcriptionally regulated by XBP1s, thereby establishing a positive feedforward loop that reinforces the IRE1α-XBP1s signaling axis in breast cancer.

**Fig. 4:**
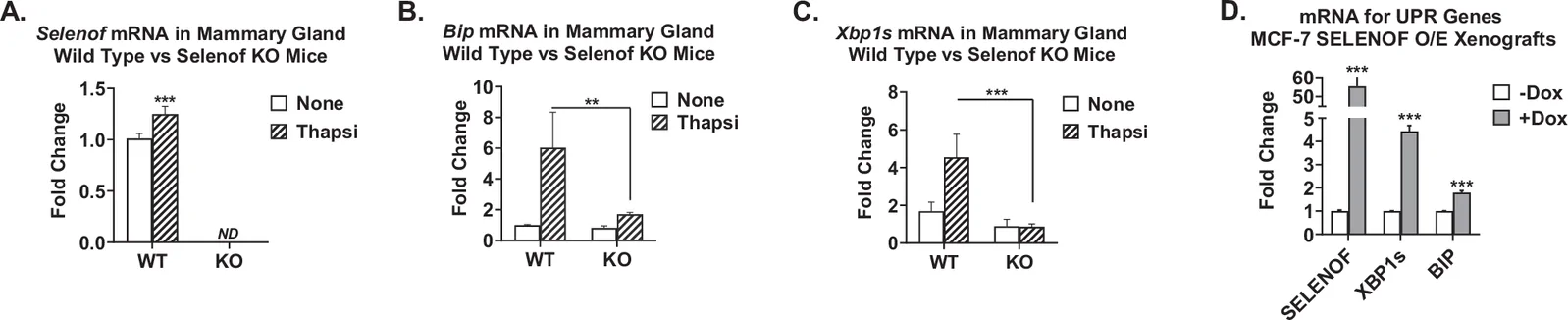
SELENOF expression is enhanced with ER stress and is required to activate UPR in murine mammary glands. **A**–**C** *Selenof* (**A**) *Bip* (**B**) and *Xbp1s* (**C**) mRNA expression in the mammary glands of wild type (WT) or Selenof knockout (KO) female mice. Mice were treated with or without 0.8 μg/g thapsigargin (Thapsi) for 16 h. **D** *SELENOF*, *XBP1s* and *BIP* mRNA expressions were determined in xenografts established from MCF-7 SELENOF cells in mice treated with or without doxycycline in drinking water as previously reported [4]. Data represents the mean ± SEM of independent experiments. **P ≤ 0.01, ***P ≤ 0.001, ND is not detected.

To determine whether crosstalk between SELENOF and the IRE1α-XBP1s branch of the UPR occurs in vivo, we treated 7-week-old female Selenof wild-type (WT) and whole-body knockout (KO) mice with thapsigargin or vehicle control. Thapsigargin (0.8 μg/g, body weight) was administered via intraperitoneal injection for 16 h, as previously described [20]. Following treatment, murine mammary glands and liver tissues were collected for total RNA extraction and gene expression analysis by RT-QPCR. *Selenof* KO status was confirmed by genotyping (Supplementary Fig. 4A). In WT mice, thapsigargin significantly induced *Selenof* mRNA expression in mammary glands, whereas no expression was detected in KO tissues (Fig. 4A). Notably, loss of Selenof markedly blunted the thapsigargin-induced upregulation of *Bip* and *Xbp1s* returning their expression to baseline levels (Fig. 4B, C). Similarly, the *Perk-Atf4-Gadd34* axis was also attenuated in the absence of Selenof (Supplementary Fig. 4B–D), indicating a broader attenuation in UPR signaling in the murine mammary gland. In previously reported MCF-7 xenograft tumors with or without SELENOF overexpression [4], we observed a significant corresponding increase in *XBP1s* and *BIP* mRNA expression (Fig. 4D), along with a more modest upregulation of on the *PERK-ATF4-GADD34* axis (Supplementary Fig. 4E). These in vivo findings in the mammary gland are consistent with our in vitro observations in breast epithelial cells, reinforcing the role of SELENOF as a critical amplifier of the UPR stress response.

Thapsigargin induced Selenof mRNA expression in the mouse liver by 1.8-fold (Supplementary Fig. 5A), consistent with the increase observed in the mammary gland. However, in the liver, loss of Selenof exacerbated UPR, as evidenced by increased *Xbp1s* (Supplementary Fig. 5B) and *Bip* (Supplementary Fig. 5C) expression, whereas other ER stress mediators, including *Perk*, *Atf4*, and *Gadd34*, were largely unaffected (Supplementary Fig. 5D–F). Together, these results indicate that ER stress upregulates SELENOF in multiple tissues, but that loss of SELENOF uniquely attenuates ER stress in the mammary gland.

### SELENOF promotes cellular senescence induced by ER stress

Given that SELENOF amplifies the UPR in breast epithelial cells, we next examined the cellular consequences of this response. Although prolonged or excessive ER stress can trigger apoptosis [21], the concentrations of thapsigargin used to induce a robust UPR in a SELENOF-dependent manner (5 nM–1 μM) did not cause significant apoptosis in MCF-10A cells, as determined by annexin V and propidium iodide staining via flow cytometry (Supplementary Fig. 6A). Notably, much higher concentrations of thapsigargin (4–16 μM) are required to induce MCF-10A cell death.

**Fig. 5:**
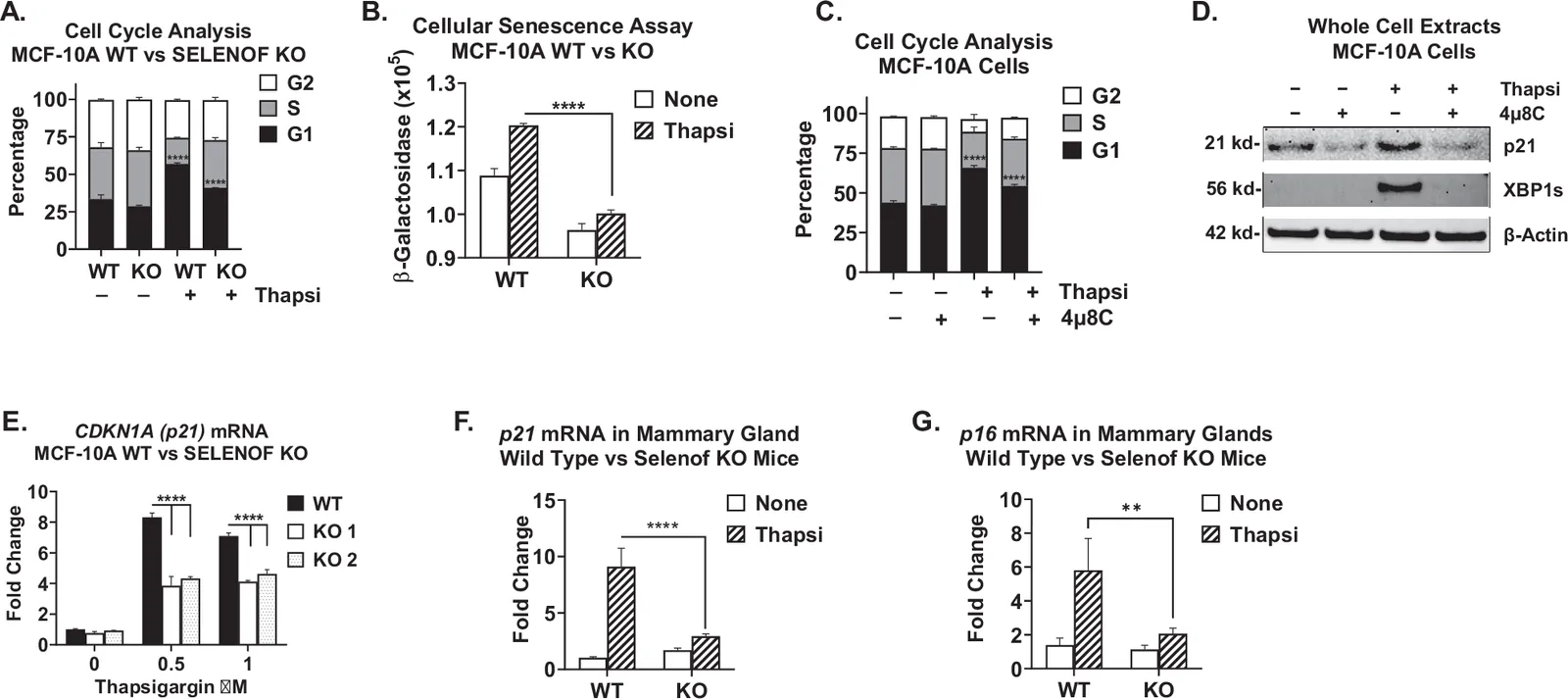
SELENOF’s regulation of UPR induces cellular senescence in the breast epithelium. **A** Cell cycle distribution was determined by FACS in MCF-10A WT cells or SELENOF KO cells were treated with or without 5 nM thapsigargin for 24 h. Statistical significance for the G1 phase is indicated. **B** SA-β-galactosidase activity (mean fluorescence intensity) was determined by flow cytometry in MCF-10A WT and SELENOF KO cells treated with 5 nM thapsigargin for 72 h. **C** Cell cycle analysis of MCF-10A WT cells and SELENOF KO cells treated with or without 5 nM thapsigargin and 5 μM 4μ8C inhibitor for 24 h. **D** p21 and XBP1s levels were determined in MCF-10A cells treated with or without 5 nM thapsigargin and 5 μM 4μ8C inhibitor for 6 h. **E** *CDKN1A (p21)* mRNA expression was determined in MCF-10A WT cells and SELENOF KO cells treated with thapsigargin for 24 h. **F**, **G** *Cdkn1a (p21)* and *Cdkn2a (p16)* mRNA expressions in the mammary glands from Selenof WT or KO mice treated with or without 0.8 μg/g thapsigargin for 16 h. Data represents the mean ± SEM. **P ≤ 0.01, ****P ≤ 0.0001.

Instead, thapsigargin induced a pronounced G1-phase cell cycle arrest, with the G1 population increasing from 33% in control cells to 57% following treatment, accompanied by a reduction in S-phase cells from 35% to 18% (Fig. 5A, 1^st^ vs 3 ^rd^ bars). This effect was SELENOF-dependent, as SELENOF KO cells exhibited a significantly attenuated G1-phase expansion under the same conditions from 29% in control cells to only 41% following treatment (Fig. 5A, 2^nd^ vs 4^th^ bars). These cells also displayed a senescent-like phenotype, evidenced by increased senescence-associated β-galactosidase (SA-βGal) staining, a widely used biomarker of cellular senescence (Fig. 5B) [22]. This senescence marker was notably reduced in SELENOF KO cells as detected by flow cytometry (Fig. 5B) and microscopy (Supplementary Fig. 7A). Thapsigargin-induced G1 arrest also appeared to be IRE1α-dependent, as treatment with the IRE1α inhibitor 4µ8C reversed the phenotype (Fig. 5C). Together, these data indicate that intact SELENOF is required to amplify the IRE1α-XBP1s axis under ER stress and to induce senescence in MCF-10A cells.

We have previously reported that SELENOF modulates the levels of cyclin-dependent kinase inhibitors p21 and p27 [5], with p21 serving as a critical regulator of cellular senescence. Consistent with this, we observed that p21 levels increase in response to thapsigargin-induced ER stress in an IRE1α-dependent manner, as treatment with the IRE1α inhibitor reversed this upregulation (Fig. 5D). Furthermore, the ER stress-dependent regulation of p21 requires SELENOF, as SELENOF loss significantly attenuated their expression in both MCF-10A cells and the mouse mammary gland (Fig. 5E, F). A similar pattern was observed for p16 in mammary glands (Fig. 5G); however, p16 expression was below the detection limit in MCF-10A cells.

**Fig. 6:**
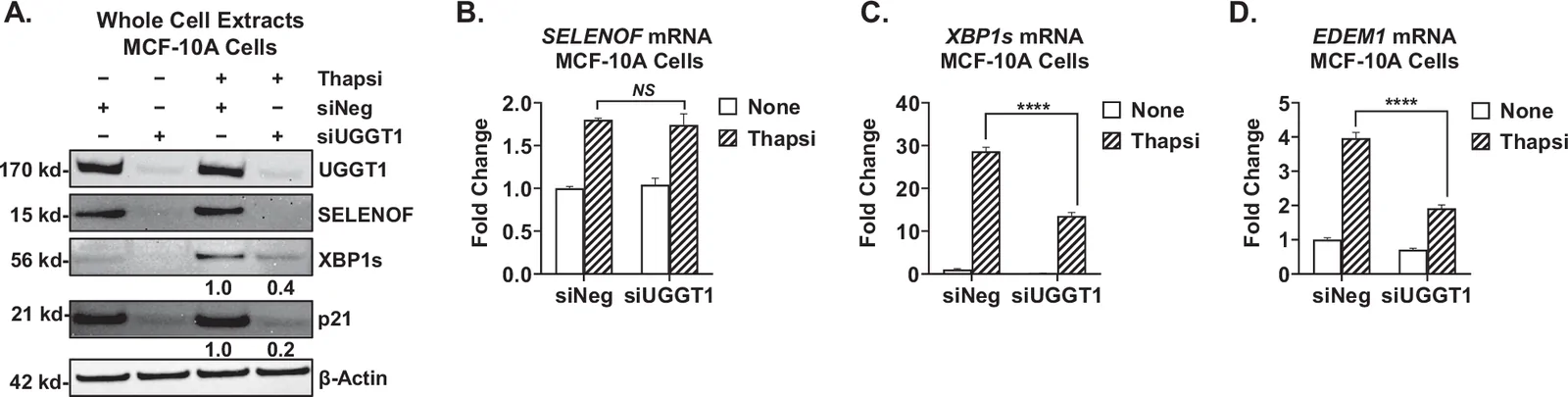
Silencing UGGT1 phenocopies the loss of SELENOF effect on UPR. **A** UGGT1, XBP1s, p21, and SELENOF levels were determined in MCF-10A cells transfected with either siNeg or siUGGT1, 10 nM each for 48 h, followed by 1 μM thapsigargin treatment for 24 h. Protein densitometry values are shown below the bands. **B**–**D** *SELENOF* (**B**), *XBP1s* and *EDEM1* mRNA expressions were determined by RT-QPCR in MCF-10A cells treated as in (**A**). Data represents the mean ± SEM of the independent experiments. ****P ≤ 0.0001. NS is not significant.

Given that the only experimentally validated interactor of SELENOF is UGGT1 [23], we examined its contribution to SELENOF regulation and its UPR-related phenotype. Silencing UGGT1 with siRNA in MCF-10A cells completely depleted SELENOF protein levels (Fig. 6A, 1^st^ vs 2^nd^ lanes) without affecting SELENOF mRNA expression (Fig. 6B), indicating post-transcriptional regulation. Importantly, UGGT1 silencing phenocopied SELENOF loss with respect to thapsigargin-induced IRE1α-XBP1s signaling, as evidenced by a 60% reduction in XBP1s protein levels and an approximately 50% decrease in *XBP1s* and *EDEM1* mRNA expression (Fig. 6C, D). While the full biological consequences of UGGT1 silencing in MCF-10A cells remain to be determined, the observed 80% reduction in p21 levels (Fig. 6A) suggests that UGGT1 may also phenocopy SELENOF loss in regulating ER stress-driven cellular senescence.

**Fig. 7:**
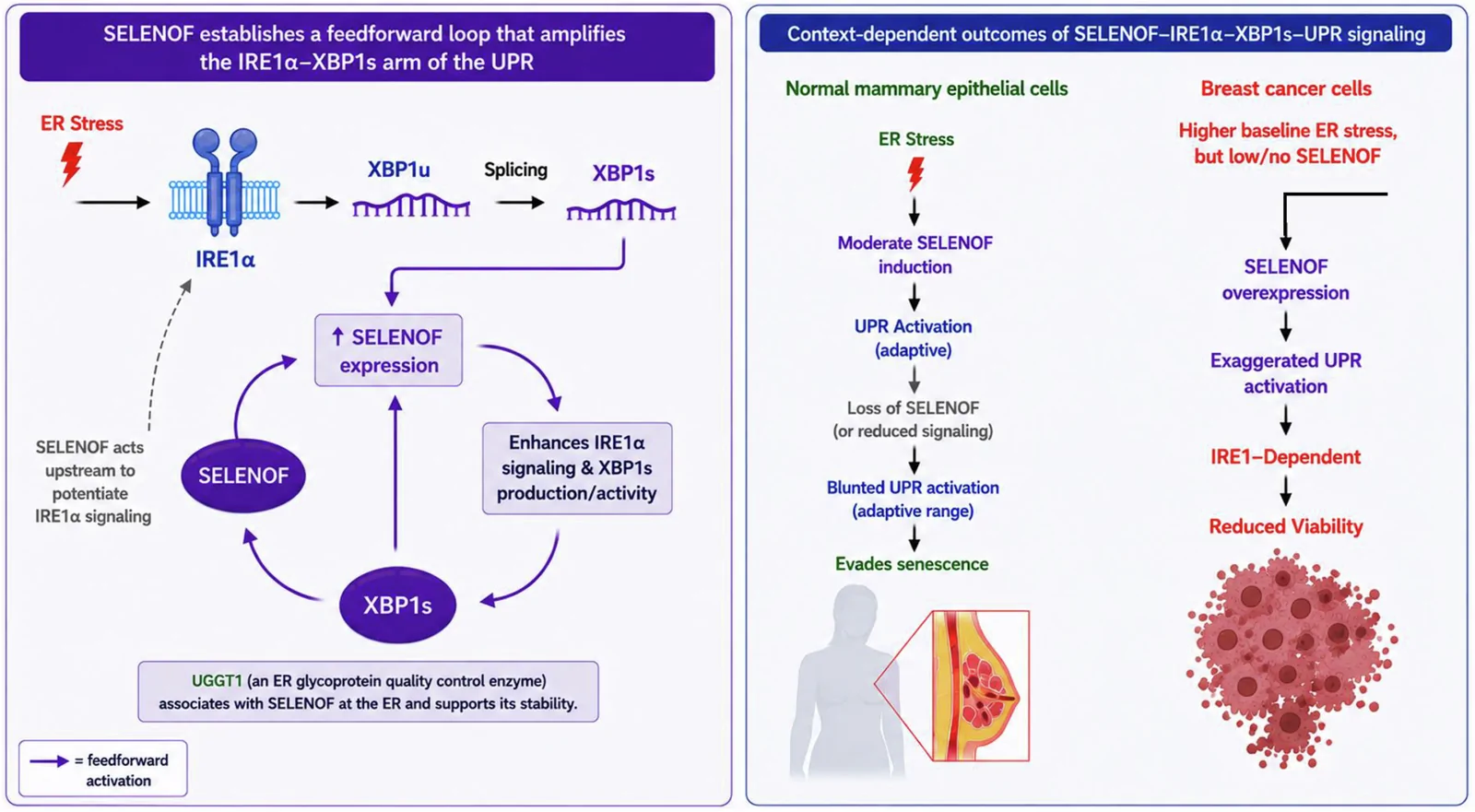
SELENOF forms a feed forward loop with the IRE1-XPB1 arm of the UPR to dictate cellular outcomes in breast epithelial cells. Left: SELENOF functions as a critical modulator of the IRE1-XBP1 arm of the UPR, while SELENOF expression itself is induced by ER stress via an IRE1-XBP1s-dependent mechanism. Right: In normal breast epithelium, an ER stress-induced IRE1α-XBP1s-SELENOF feedforward loop reinforces senescence, whereas loss of SELENOF enables cells to evade this response. In breast cancer cells, low or absent SELENOF expression supports cell growth and viability despite elevated basal ER stress. Conversely, SELENOF overexpression exacerbates UPR activation and reduces cell viability in an IRE1α-dependent manner.

Together, these findings indicate that SELENOF is essential for amplifying the IRE1α-XBP1s axis, thereby promoting cell cycle arrest and senescence in response to ER stress. Conversely, loss of SELENOF disrupts this regulatory mechanism, enabling cells to continue surviving despite persistent ER stress (Fig. 7).

## Discussion

Based on our previous findings that SELENOF overexpression in breast cancer cells elicits anti-tumor effects, whereas its loss promotes proliferation and resistance to cell death, we proposed that SELENOF likely functions as a tumor suppressor in breast cancer [4, 5]. However, our recent patient data demonstrated that SELENOF protein levels are significantly elevated in human breast tumors compared to normal tissue [24]. These findings underscore the complexity and the need for a deeper understanding of SELENOF’s role in the breast, its molecular mechanisms, and possibly its context-dependent effects. Despite limited understanding of SELENOF function in normal physiology [7], a postulated role in protein redox quality control prompted us to investigate SELENOF’s broader relationship with the UPR. In this study, we demonstrate that SELENOF is required to amplify stress-induced UPR and its downstream effectors, primarily the IRE1α-XBP1s axis, in both mammary epithelial and breast cancer cells. In a feedforward loop, ER stress enhances SELENOF expression via IRE1α-XBP1s signaling. Importantly, SELENOF’s impact on the UPR induces cellular senescence, characterized by G1 cell cycle arrest, increased p21 expression, and SA-βGal activity.

The context-specific nature of the UPR in breast cancer underscores the importance of understanding the contribution of each UPR arm individually. In our models, SELENOF predominantly influenced the IRE1α-XBP1s axis. Beyond *XBP1* mRNA splicing, IRE1α also mediates regulated IRE1α-dependent decay (RIDD) of ER-localized mRNAs. How these dual RNase outputs function in early cancer stages remains unclear. In epidermal keratinocytes, the epithelial cells of the skin, the IRE1α pathway is a critical downstream target of HRAS signaling, with its two RNase outputs exerting opposing effects on cell fate: XBP1 splicing enhances HRAS-driven proliferation, whereas RIDD promotes premature senescence through degradation of the pro-oncogenic factor *ID1* mRNA [25]. This illustrates the complex, context-dependent nature of IRE1α signaling in cancer, which is further compounded by the paradoxical role of senescence itself, capable of acting as both a tumor-suppressive barrier and, under certain conditions, a tumor-promoting state [26]. Contemporary views define senescence not as a fixed set of traits, but as a cellular state characterized by activation of adaptive stress pathways, including ER stress and UPR [27]. Our findings that SELENOF mediates UPR and cellular senescence in normal breast epithelial cells, and the potential implications for breast cancer, warrant further investigation. Although the importance of ER stress and the UPR for cancer initiation is not a new concept, the emerging complexity of the IRE1α pathway, SELENOF and senescence, highlights that much remains to be understood.

In cancer cells, it is generally accepted that a certain level of UPR activation is already present to meet the high proteostatic demands of tumor growth. Consistent with this, IRE1α’s RNase activity has been reported to be constitutively active, particularly in basal-like/TNBC breast cancer cells, where it contributes to the production of pro-tumorigenic factors and promotes tumorigenesis [28]. However, activation of the IRE1α-XBP1s signaling axis does not always create an adaptive environment that favors cell growth. Under persistent stress, IRE1α’s output can shift toward pro-apoptotic signaling through the TRAF2-JNK pathway [29]. In such contexts, inhibition of IRE1α RNase activity has been shown to promote cell survival under severe ER stress [30]. Moreover, the IRE1α-XBP1s pathway exhibits complex roles in balancing growth control and cancer progression. For example, IRE1α-XBP1s activation has been shown to upregulate BIP sequestering YAP (Yorkie in *Drosophila*) to limit proliferation and regulate organ size. Unexpectedly, hyperactivation of IRE1α-XBP1s signaling in TNBC cells also induced JNK-dependent cell migration and epithelial-mesenchymal transition (EMT) via repression of YAP [31]. In our study, pharmacological inhibition of IRE1α mitigated the ER stress induced by SELENOF overexpression and reversed its growth-inhibitory effect in breast cancer cells. While this output would be beneficial against cancer growth, it remains to be determined whether such inhibition might also promote other aggressive phenotypes.

Not surprisingly, like many proteins that participate in UPR, we found that ER stress upregulates SELENOF expression in vitro and in the mammary gland in vivo, consistent with prior reports in murine fibroblasts where various ER stressors similarly modulated SELENOF levels [12]. We found that induction of SELENOF was regulated at the transcription level through XBP1s activated by IRE1α. The observation that SELENOF levels are elevated in breast tumors compared to adjacent normal tissue [24] is consistent with the notion that tumors experience a heightened ER stress burden, which may in turn drive upregulation of SELENOF expression. One possibility is that XBP1s directly binds to the SELENOF promoter. Alternatively, XBP1s may act indirectly by regulating another transcription factor, such as heat shock factor 1 (HSF1), which is upregulated during ER stress [32]. HSF1, in turn, has been reported to bind the SELENOF promoter, thereby modulating *SELENOF* mRNA expression [33]. In the mouse mammary gland, SELENOF deficiency attenuated not only Ire1α-Xbp1s signaling but also the Perk-Atf4-Chop axis. Prior studies have suggested that IRE1α-XBP1s ablation can reduce PERK signaling in breast cancer cells [34], highlighting potential crosstalk that warrants further investigation at additional time points in vivo.

One-third of the human proteome traverses the ER where folding and maturation are facilitated by a complex protein network that involves both glycan modifications and disulfide bond formation; the SELENOF-UGGT1 complex is of key importance here. SELENOF is proposed to act as a redox co-chaperone that enhances UGGT1’s ability to sense and facilitate the proper folding of misfolded proteins, although its exact function in this partnership remains largely a mystery due to lack of structural data [23]. A recently predicted structure of the SELENOF-UGGT1 complex positions the selenocysteine residue of SELENOF in proximity to the UGGT1 catalytic site [23], lending support to its proposed co-chaperone role. Our findings suggest a reciprocal influence of UGGT1 on SELENOF’s function in the UPR. While cooperative interactions between the two proteins are plausible, this reciprocal effect is likely mediated through UGGT1’s impact on SELENOF stability.

Most of our data examining the role of SELENOF was generated using CRISPR/Cas9-mediated knockout of SELENOF in MCF-10A cells, with additional validation by siRNA-mediated knockdown and in vivo corroboration in Selenof KO mice. Given the focus on breast cancer, only female mice were used, which represents a limitation in generalizing the impact of SELENOF on the UPR across both sexes. The contribution of individual UPR arms and their downstream signaling pathways was interrogated using siRNAs and, when available, pharmacological inhibitors. In vivo, limited tissue availability restricted our analyses to gene expression measurements without corresponding protein-level validation, representing an additional limitation. Lastly, two distinct ER stress inducers, thapsigargin and tunicamycin, were employed in normal MCF-10A cells as well as in a panel of breast cancer cell lines to ensure robustness and reproducibility of the findings.

Collectively, our study highlights a critical role for SELENOF in regulating ER stress response and associated induction of cellular senescence (Fig. 7). Loss of SELENOF may constrain UPR activation to adaptive levels, thereby enabling cells to evade senescence and acquire a selective advantage during oncogenic transformation. Conversely, strategies aimed at restoring SELENOF expression or pharmacologically enhancing the SELENOF-IRE1α-XBP1s signaling axis may hold potential for anti-breast cancer therapy. Nonetheless, given the multifaceted and context-dependent roles of the IRE1α pathway and cellular senescence in cancer, these interpretations and therapeutic possibilities should be pursued with careful consideration.

## Methods and Materials

### Reagents

Doxycycline (Dox), 4’,6-diamidino-2-phenylindole (DAPI), IRE1α inhibitors (STF083010, 4µ8C), PERK inhibitor (GSK2606414), ATF6 inhibitor (CEAPIN-A7), sodium selenite, thapsigargin, tunicamycin, and thioflavin T were purchased from Sigma (St. Louis, MO, USA), Invitrogen (Waltham, MA, USA), Thermo Fisher Scientific (Waltham, MA, USA), Selleck Chemicals (Houston, TX, USA), and APExBIO (Houston, TX, USA). Antibodies specific to SELENOF (ab124840), XBP1s (ab220783), IRE1α (sc-390960), CHOP (2895 T), BIP (66574-1-Ig), UGGT1 (sc-374565) and β-actin (A5441) were respectively purchased from Abcam (Boston, MA, USA), Santa Cruz (Dallas, TX, USA), Cell Signaling Technology (Boston, MA, USA) and Sigma. siRNA for SELENOF (s17999), siRNA for IRE1α (SC-270028), siRNA for XBP1 (SC-38627), and siRNA for UGGT1 (HSS183580) were purchased from Thermo Fisher Scientific and Santa Cruz (Dallas, TX, USA).

### Cell lines, culture conditions and treatments

MCF-10A, MCF-7, MDA-MB-231, HCC-1806 cells were purchased from ATCC. The cells were maintained at 37 °C in a humidified atmosphere with 5% CO_2_, and the media were changed every 2–3 days. MCF-10A SELENOF knockout (KO) cells were generated using CRISPR-Cas9 genome editing as previously reported [5]. SELENOF knockout efficiency was determined by western blotting. MCF-10A or SELENOF KO clones were maintained in DMEM-F12 medium (Gibco, Waltham, MA, USA) supplemented with 10% fetal bovine serum (Gibco), 20 ng/mL epithelial growth factor, 0.5 mg/mL hydrocortisone, 100 ng/mL cholera toxin, and 10 µg/mL insulin. MCF-7 cells overexpressing SELENOF (MCF-7 SELENOF cells) were generated through stable transfection of MCF-7 cells with a Dox-inducible SELENOF expression construct, pRetroX-Tight-Pur-TetOn-Advanced, from Clontech, as previously reported [4]. MCF-7 cells and MCF-7 SELENOF cells were maintained in RPMI 1640 (Gibco) medium with phenol red supplemented with 10% fetal bovine serum, 1% non-essential amino acids, 2 mmol/L L-glutamine, 1% penicillin–streptomycin antibiotics, 6 ng/mL insulin, and selection antibiotics (250 µg/mL geneticin and 1 µg/mL puromycin). To induce SELENOF expression, cells were treated with 1 µg/mL Dox. MDA-MB-231 cells were maintained in RPMI 1640 medium (Gibco) supplemented with 5% fetal bovine serum (FBS), 2 mmol/L L-glutamine, 1% antibiotics penicillin-streptomycin, and 1% non-essential amino acids. HCC1806 cells were obtained from ATCC and maintained in RPMI 1640 medium with HEPES and sodium pyruvate (ATCC, 30-2001) supplemented with 10% FBS, 2 mmol/L L-glutamine, 1% antibiotics penicillin-streptomycin, and 1% non-essential amino acids. All cell lines are routinely tested for mycoplasma using the LookOut PCR detection kit from Sigma.

### Cell viability

Cells were seeded in 24-well plates, and medium was replenished every 2–3 days. Cells were stained with 1% crystal violet in methanol and water (1:4) and solubilized in 1% sodium dodecyl sulfate. The absorbance was measured at 570 nm using a Polarstar Omega fluorescence plate reader (BMG Labtech, Ortenberg, Germany).

### RT-Quantitative PCR (QPCR)

Total RNA was isolated using TRIzol according to manufacturer’s instructions. RNA (0.5 µg) was reverse transcribed in a total volume of 10 μL using 200U of M-MLV reverse transcriptase (0.5 μL), 100 ng of random hexamer (0.5 μL), 0.5 mM of deoxy-NTP (0.5 μL), and 100 mM of DTT (1 μL), DEPC-treated water (0.5 μL), 5X buffer (2 μL). The resulting cDNA was diluted with 90 µL of DEPC-treated water, then 2 μL cDNA were mixed with SYBR Green master mix (8 μL, 5 μL 2X SYBR green, 1 μL 1.25 μM forward and reverses Primer Mix, 2 μL DEPC-treated water). Amplifications were performed using the QuantStudio3 instrument (ThermoFisher Scientific) according to the manufacturer’s instructions. The fold-change was calculated using the ΔΔCt method, with GAPDH serving as the internal control. All QPCR primers used were validated and are available upon request.

### SiRNA transfection

Cells were seeded into 6-well plates and transfected either an siRNA, 10 nM, against the target of interest or with an siNeg non-targeting siRNA control using Lipofectamine™ RNAiMAX Transfection Reagent (Invitrogen). Silencing efficiency was confirmed 24–72 h later by RT-QPCR and western blotting.

### Western blot

Whole cell extracts were prepared using the M-PER reagent (ThermoFisher Scientific). Proteins were separated using SDS-PAGE (Invitrogen), transferred to nitrocellulose membranes using the iBlot 2 instrument (Invitrogen), blocked for 1 h in TBS/T buffer containing 5% non-fat dry milk, and incubated with the appropriate primary antibody (diluted in 5% milk in TBS/T) overnight. The next day, the corresponding secondary antibody was applied for 1 h, and the signal was visualized using an iBright CL1000 Imaging System (Invitrogen) with the Pierce Supersignal West Pico chemiluminescent substrate (ThermoFisher Scientific). Protein density quantification was conducted by ImageJ and normalized to β-Actin.

### Thioflavin T staining

The cells were seeded in 24-well plates and treated as indicated. Cells were then stained with 5 µM thioflavin T solution for 1 h followed by DAPI staining (R37606) for 15 min. The images were acquired by a fluorescent microscope (Nikon, Japan) and fluorescence intensity was quantified by Image J.

### FACS analysis for cell cycle, apoptosis and senescence

For cell cycle analysis, treated cells were collected using trypsin, suspended in Hanks balanced salt solution buffer with 2% FBS, and washed twice with ice-cold PBS. The cells were then incubated with 100% cold ethanol for 60 min at 4 °C, followed by 100 μg/mL RNase and 50 μg/mL propidium iodide (PI) for 60 min, and analyzed using FACS Canto (Beckman Coulter, Brea, CA, USA). Single cells were gated and analyzed to differentiate between G1, S, and G2 phases. Apoptosis was determined using annexin V and propidium iodide (PI) staining. After treatment, cells were washed with ice-cold PBS and suspended in 100 µL of annexin binding solution containing 5 µL of annexin V-Alexa-488-conjugate (Invitrogen, V13241) and 1 µL of propidium iodide (100 µg/mL). After 15 min of incubation at room temperature in the dark, 400 µL of fresh binding buffer was added, followed by immediate analysis using the FACS Quanteon flow cytometer (Agilent Technologies, CA, USA).

For the β-galactosidase assay, cells were treated as indicated, followed by staining and analysis according to the manufacturer’s instructions (Invitrogen, C10841 and Cell signaling Technology, 9860S). Representative gating strategy for flow cytometry is included in Supplementary 6 C. For the β-galactosidase staining by microscopy, total cell number and β-galactosidase-positive cells were quantified using ImageJ.

### ER stress induction in vivo

Mouse experiments were performed at the Loyola University Chicago animal facility and conducted in accordance with institutional procedures and guidelines after prior approval from the Loyola university Chicago Institutional Animal Care and Use Committee (IACUC; Protocol NO. 2022039). 7-weeks-old female C57BL/6 mice Selenof^−/−^ (KO) and wild type mice (WT) were used. Thapsigargin was initially dissolved in dimethyl sulfoxide (DMSO) and then diluted in autoclaved PBS to obtain a final concentration of 20 mg/mL. Mice approximately 15–20 g in weight were injected intraperitoneally with the thapsigargin solution at 0.8 μg per gram of body mass. Control mice were injected intraperitoneally with a PBS solution, 50 µL. The murine mammary gland tissues (*n* = 8–12) and livers (*n* = 6–9) were harvested after 16 h of treatment, followed by total RNA extraction. Xenograft tumors with control or SELENOF overexpression were isolated as previously published, followed by total RNA extraction [4]. No formal blinding and randomization method was used. Samples were allocated sequentially; however, all experimental groups were processed in parallel under identical conditions to minimize bias. No formal statistical power calculation was performed prior to the study. Sample sizes were determined based on previous studies.

### Immunoprecipitation

Following treatment, cells were fixed with 0.5% formaldehyde for 8 min at room temperature, and the reaction was quenched with 0.1 M glycine for 5 min. Cells were lysed in MPER lysis buffer, and whole-cell lysates were collected. Ten percent of the total lysate served as input. Dynabeads Protein G (10003D) magnetic beads were incubated with the indicated antibody (4 µg) for 4 h at 4 °C with gentle rotation. After washing, the remaining lysate was added to the antibody–bead complex and incubated overnight at 4 °C with rotation. The next day, beads were resuspended in protein loading buffer and heated at 50 °C to elute bound proteins for subsequent analysis.

### Genotyping

Genotyping to confirm complete systemic loss of Selenof in our Selenof KO mice and presence of Selenof in the WT mice was done using tissue from tail snips. Tail snips of 5 mm were taken when mice were 3 weeks old. Tail snips were stored at −20 °C until tissue extraction protocol. Tissue extraction and PCR protocol was done with the REDExtract-N-AmpTM kit from Sigma-Aldrich (catalog number: XNAT-100RXN) using 25 µL of extraction solution and 6.25 µL of tissue preparation solution placed into microcentrifuge tube with tail, cut side down. Samples were then incubated for 10 min at 95 °C, after which 25 µL of Neutralization buffer was added. Samples were stored at −20 °C until REDExtract PCR protocol was performed. Primers were ordered from Integrated DNA Technologies. To detect Selenof presence, the reverse Selenof primer (TTT GGC CAG ATA CCA GGA AG) and forward Selenof primer (GCA GCT CTT GCG ATC TTC TT) were used. Since Selenof KO mice would not have a SELENOF band present, a band for the presence of the Neo cassette was used as a positive control. To detect the presence of a Neo cassette band, the Neo forward primer (TCG CCT TCT TGA CGA GTT CT) and Selenof reverse primer were used, as in Kasaikina et al. [6]. Tissue extract, REDExtract and corresponding primer mix were combined to a volume of 20 µL and placed in the thermal cycler. The samples were then run on a 1% agarose gel with SYBR Safe DNA gel stain and visualized using UV.

### RNA sequencing and bioinformatic analysis

Total RNA was extracted using TRIzol according to the manufacturer’s instructions. RNA quantity and quality were assessed using a NanoDrop spectrophotometer. RNA-seq library preparation, sequencing, and initial quality control were performed by Novogene. Libraries were sequenced using the Illumina NovaSeq platform employing a paired-end 150 bp sequencing strategy. Downstream analysis was conducted using the DESeq2 R package (1.20.0) for differential expression analysis. Gene set enrichment analysis (GSEA) was conducted using the GSEA desktop application (version 4.3.3, Broad Institute). The full gene expression dataset, quantified as FPKM (Fragments Per Kilobase of transcript per Million mapped reads), was used as input.

### Statistical analysis

The data are presented as mean ± SEM from a minimum of three independent determinations, with *P* ≤ 0.05 as a significant difference. Statistical analysis consisted of 1- or 2-way ANOVA followed by Tukey posttest, or a t-test when applicable.

## Supplementary information

Supp Fig. Legends

Supplementary Figures

Supplementary Material Original Western Blots

## Data Availability

The RNA sequencing data generated during this study are publicly available in the NCBI Sequence Read Archive (SRA) under BioProject accession PRJNA1504261. All other data supporting the findings of this study are available from the corresponding author upon reasonable request.
