# Supplementary material for: Crosstalk between SELENOF and the unfolded protein response in breast epithelium dictates cell fate
Source: Cell Death Discov. 2026 Aug 10;12:445. doi: 10.1038/s41420-026-03285-7 (PMC13740312; doi:10.1038/s41420-026-03285-7)

Supplementary Figure 1.

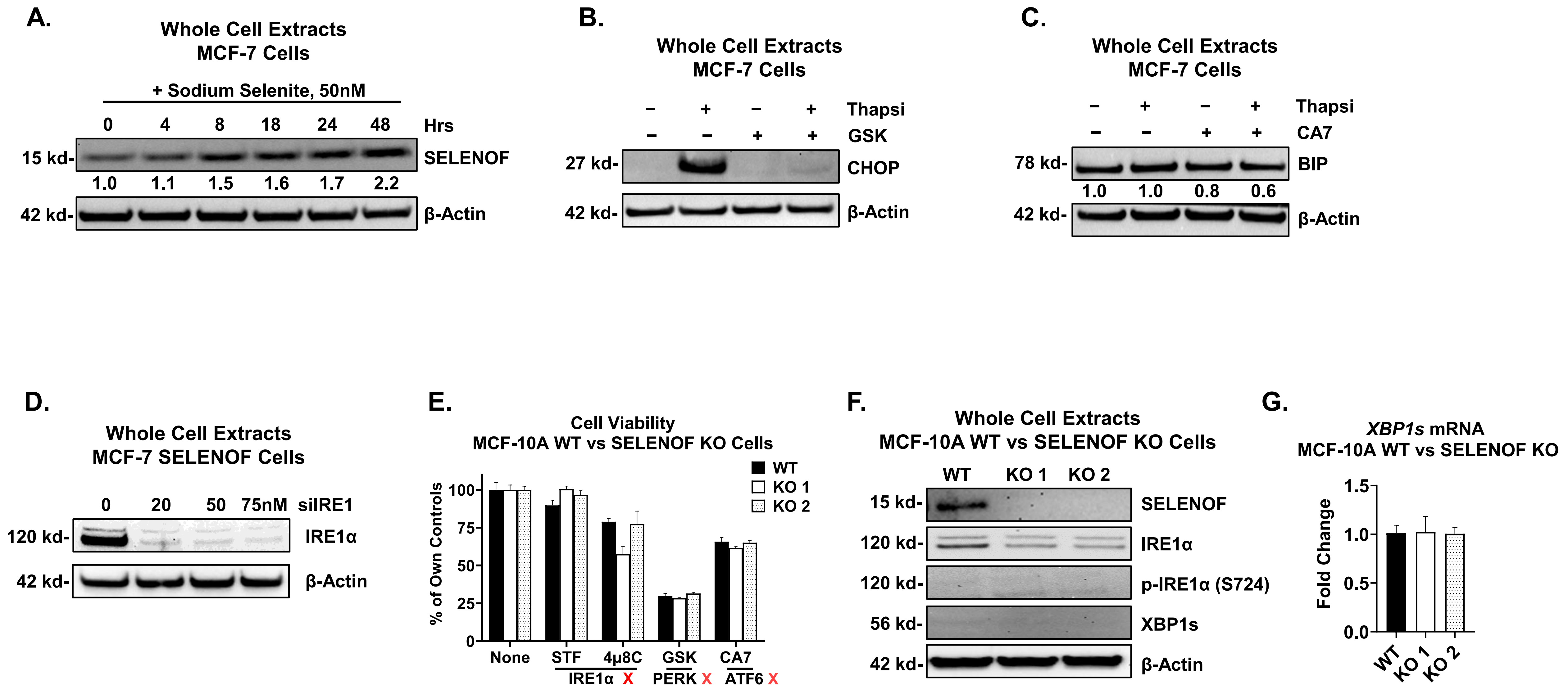

Supplementary Figure 2.

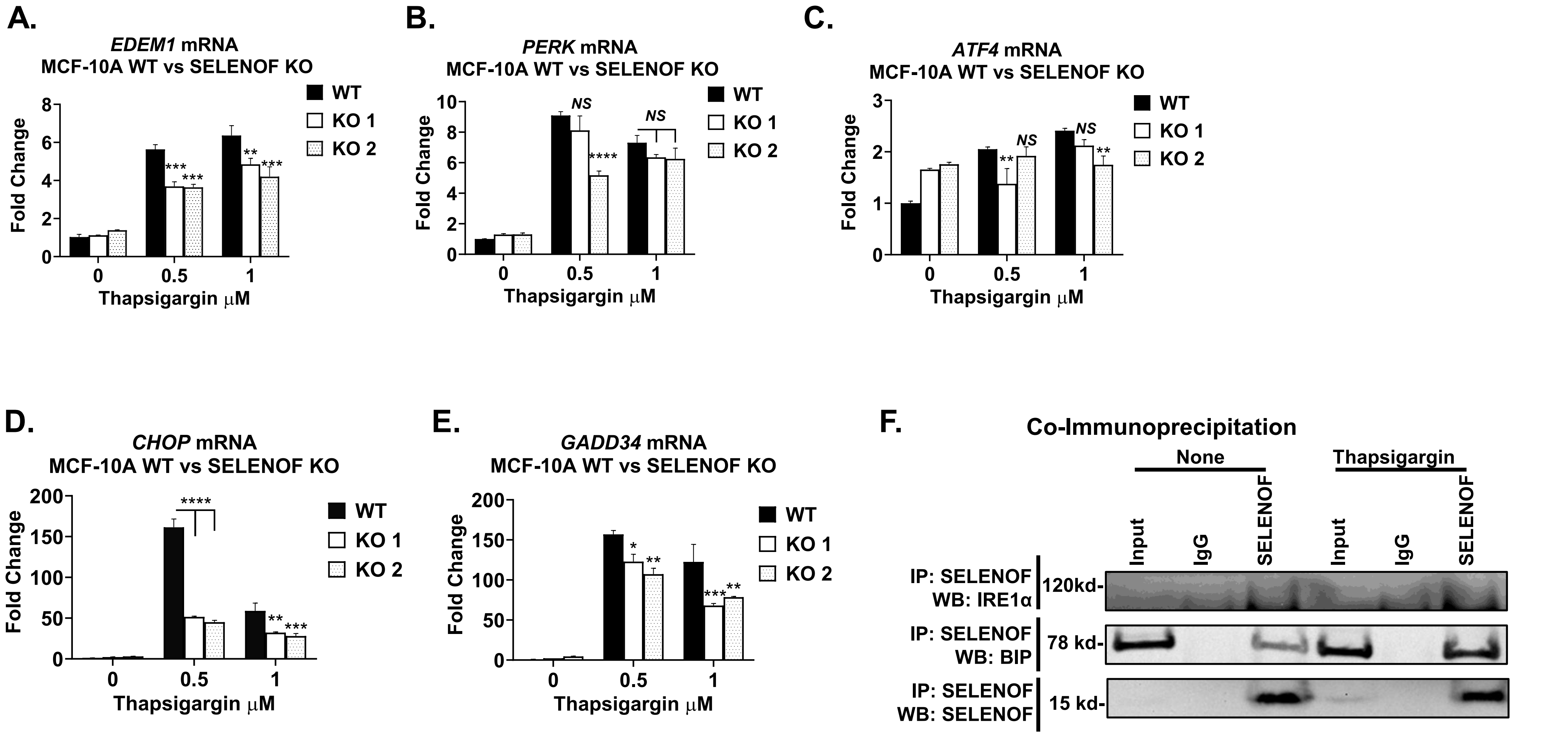

Supplementary Figure 3.

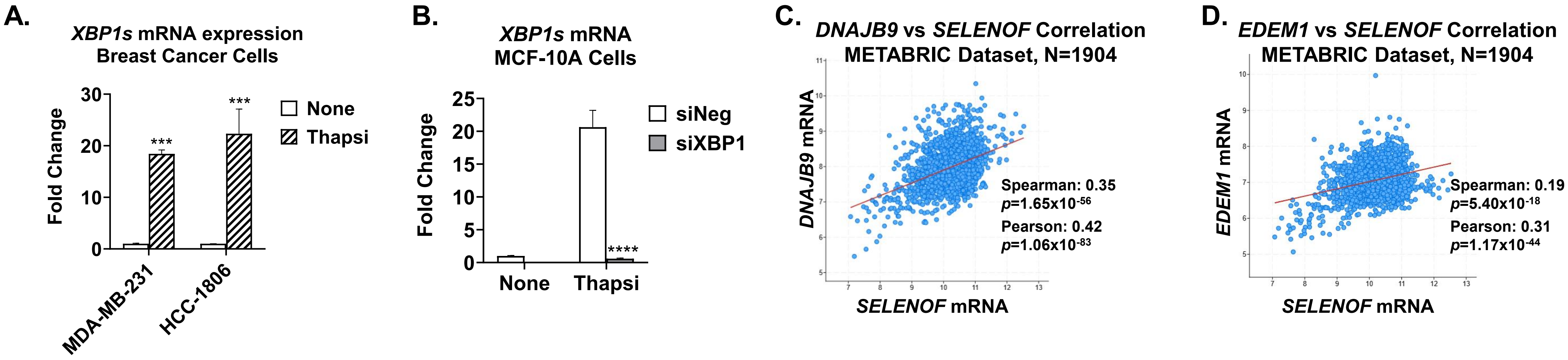

Supplementary Figure 4.

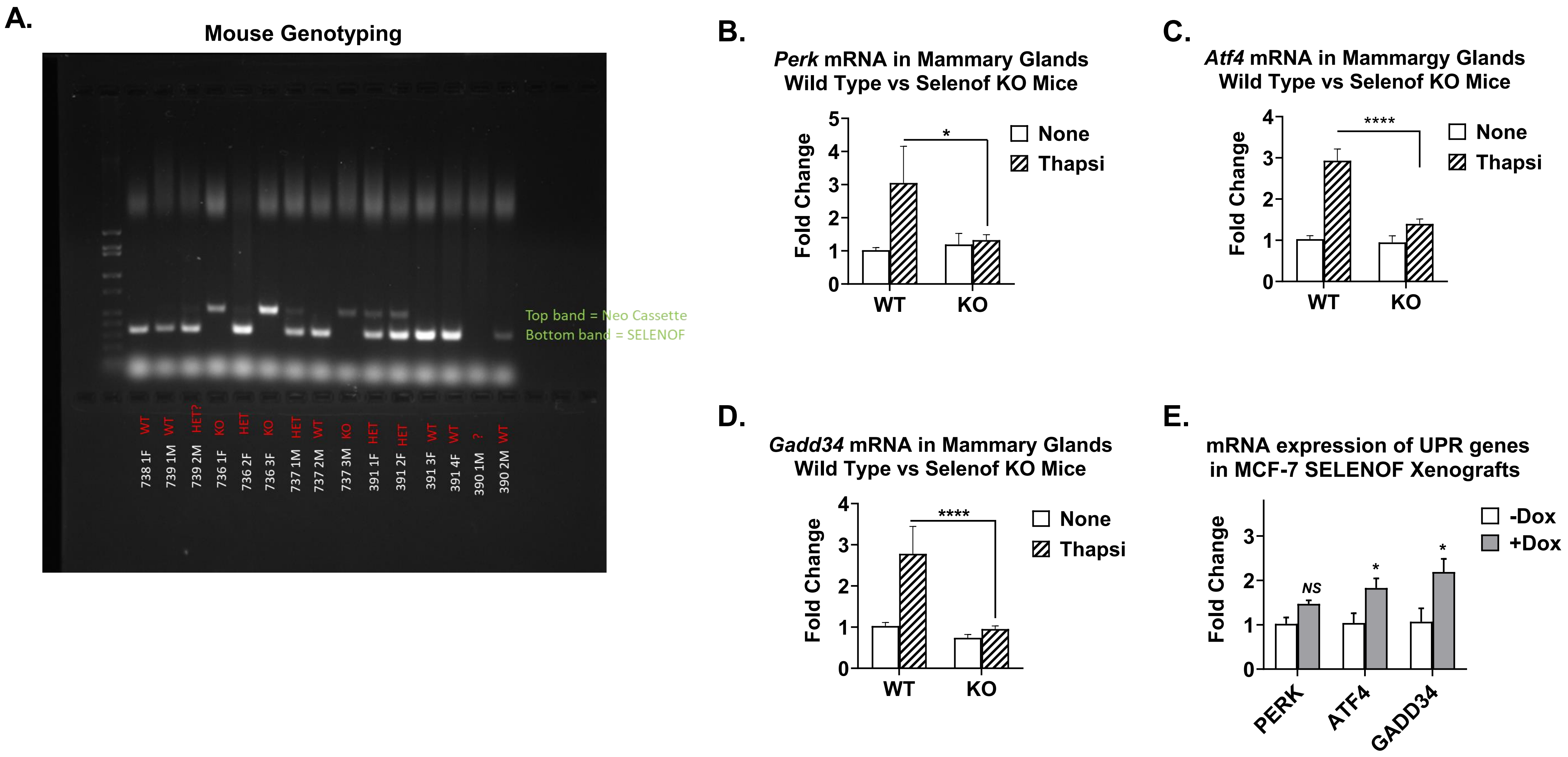

Supplementary Figure 5.

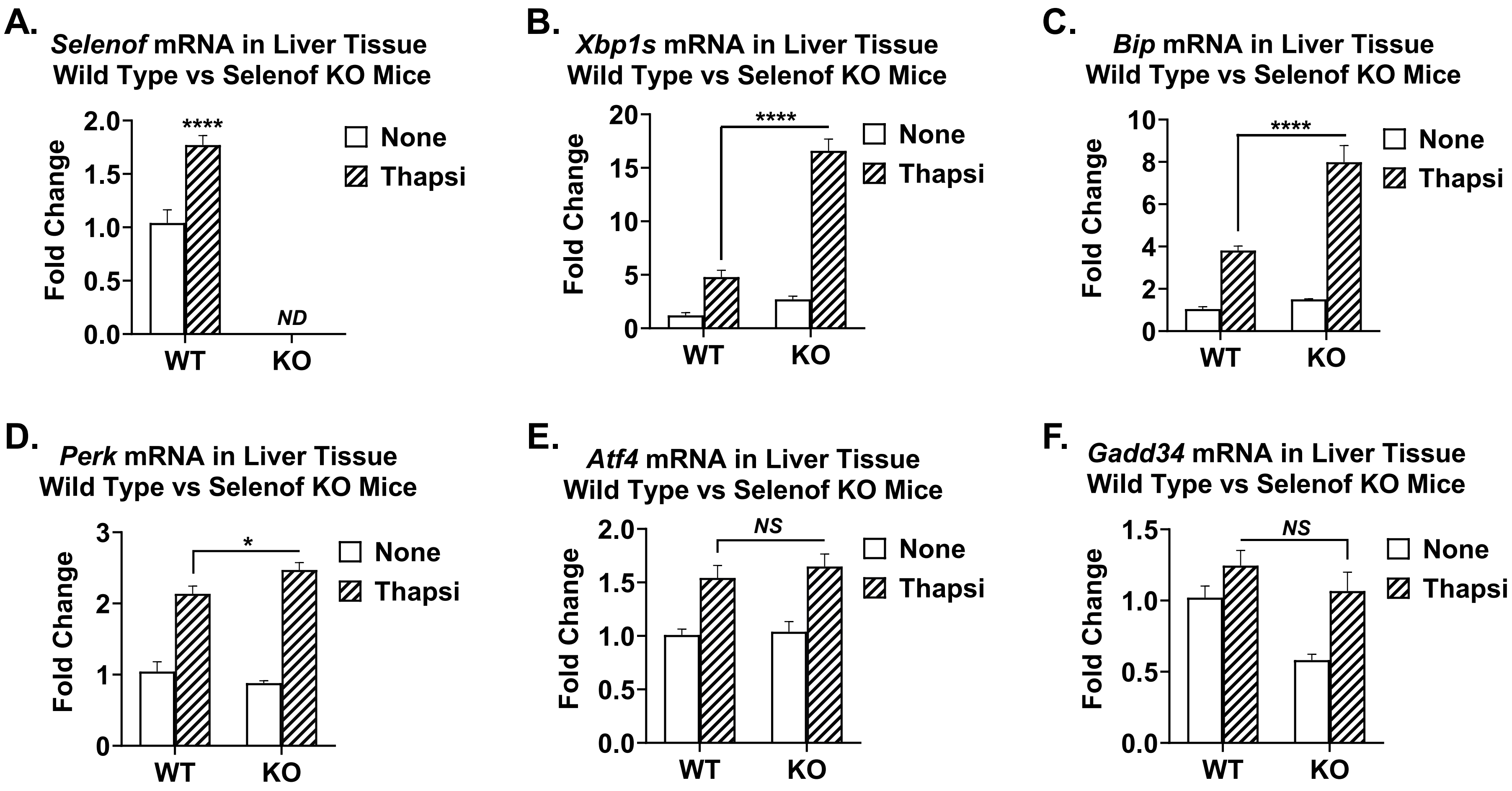

Supplementary Figure 6.

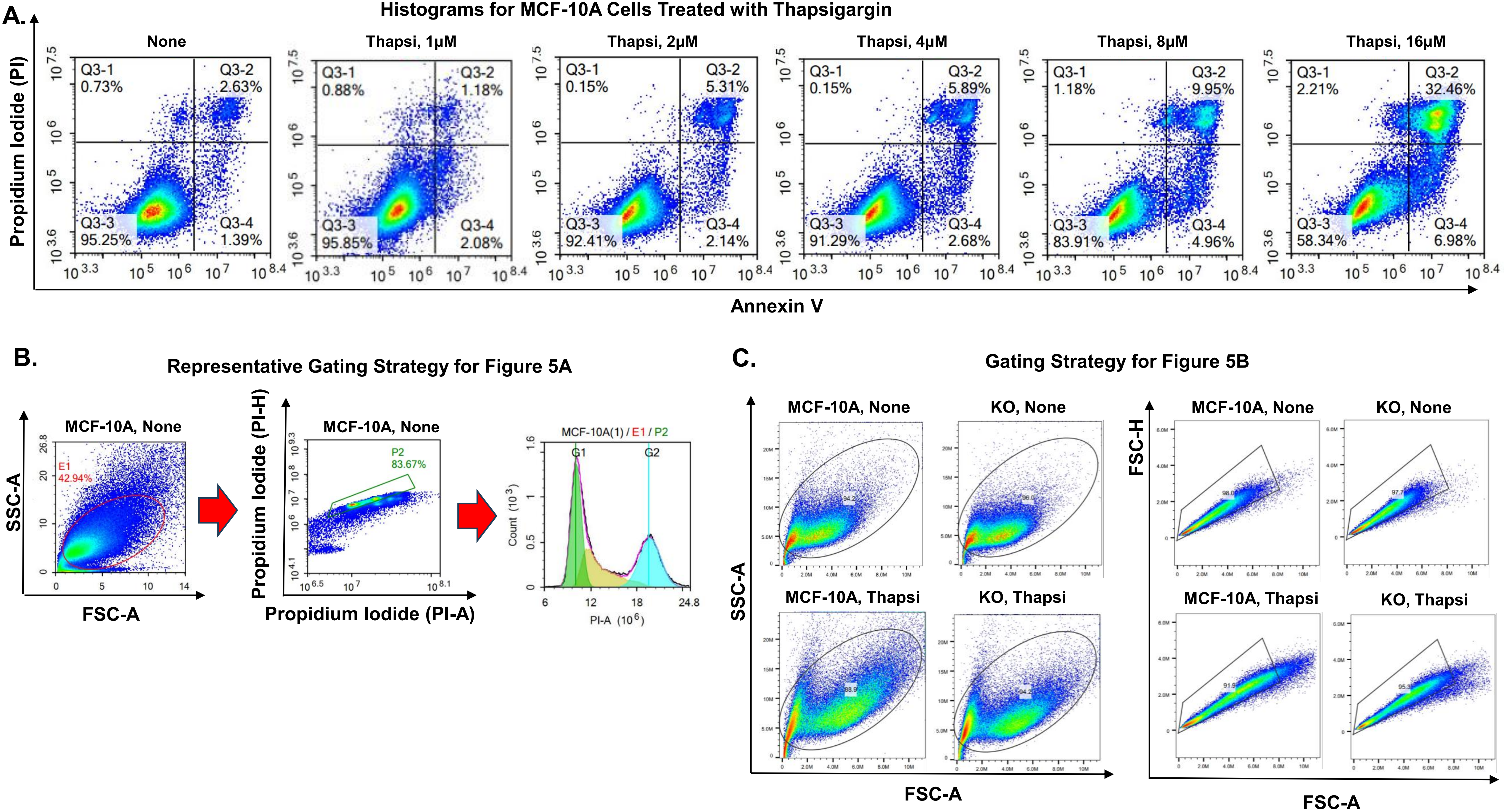

Supplementary Figure 7.

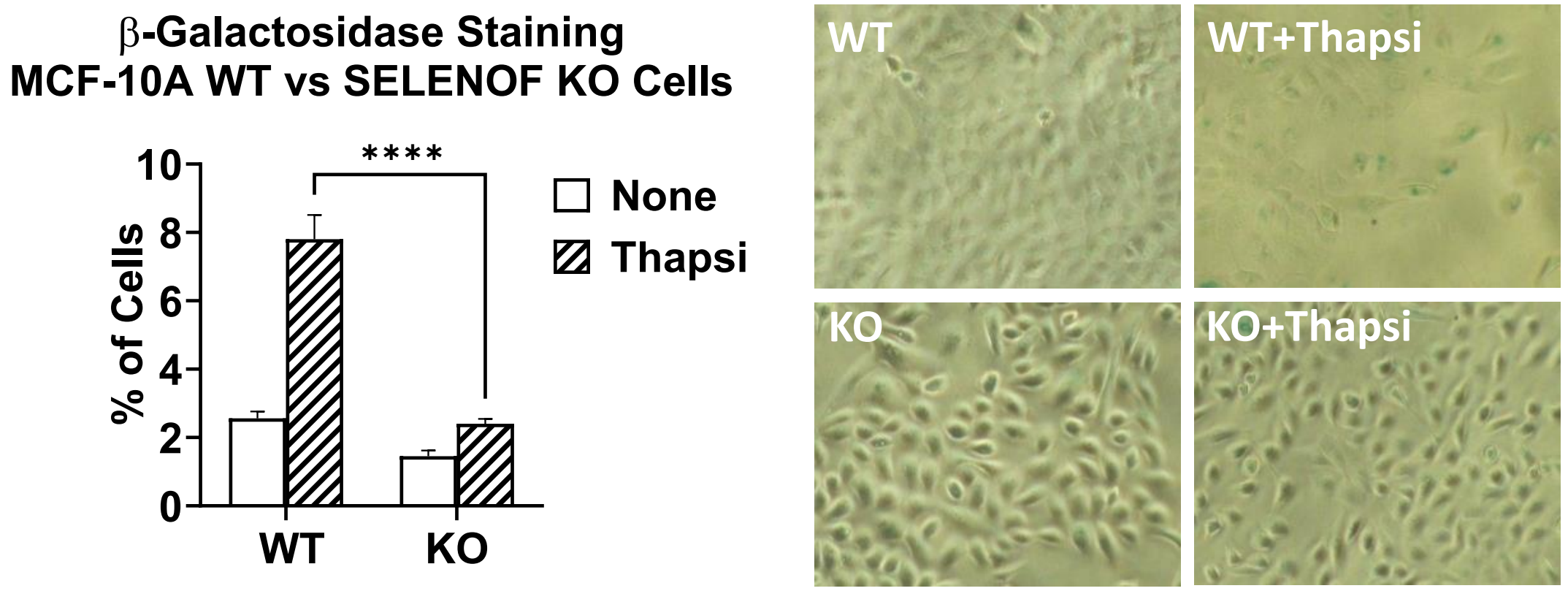

Supplement: Supplementary file 1 [file 41420_2026_3285_MOESM2_ESM.pdf]
