# Supplementary material for: Crosstalk between SELENOF and the unfolded protein response in breast epithelium dictates cell fate
Source: Cell Death Discov. 2026 Aug 10;12:445. doi: 10.1038/s41420-026-03285-7 (PMC13740312; doi:10.1038/s41420-026-03285-7)

# Original Western Blot

Figure 1.

A.

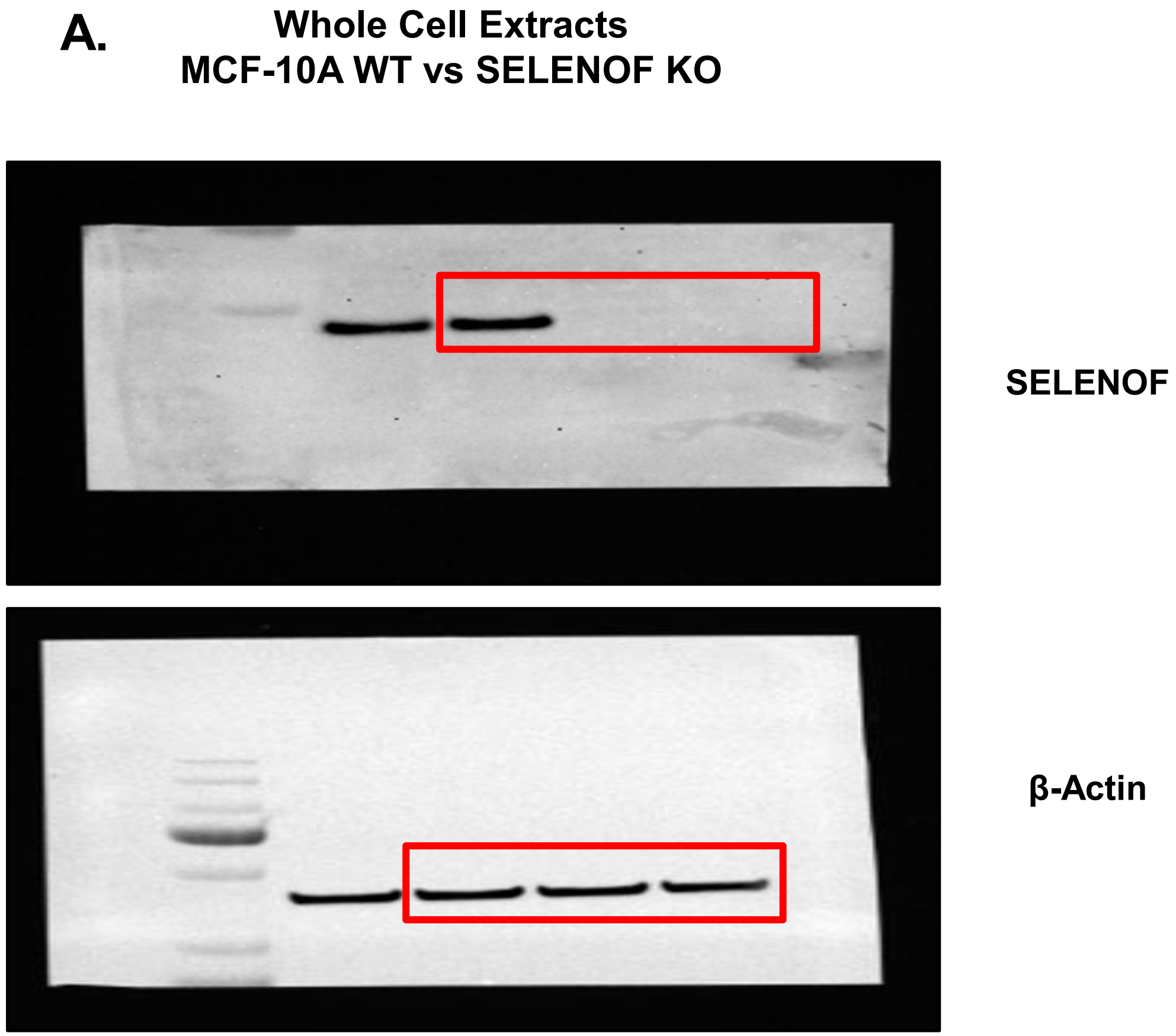

Whole Cell Extracts
MCF-10A WT vs SELENOF KO
SELENOF
β-Actin


C.

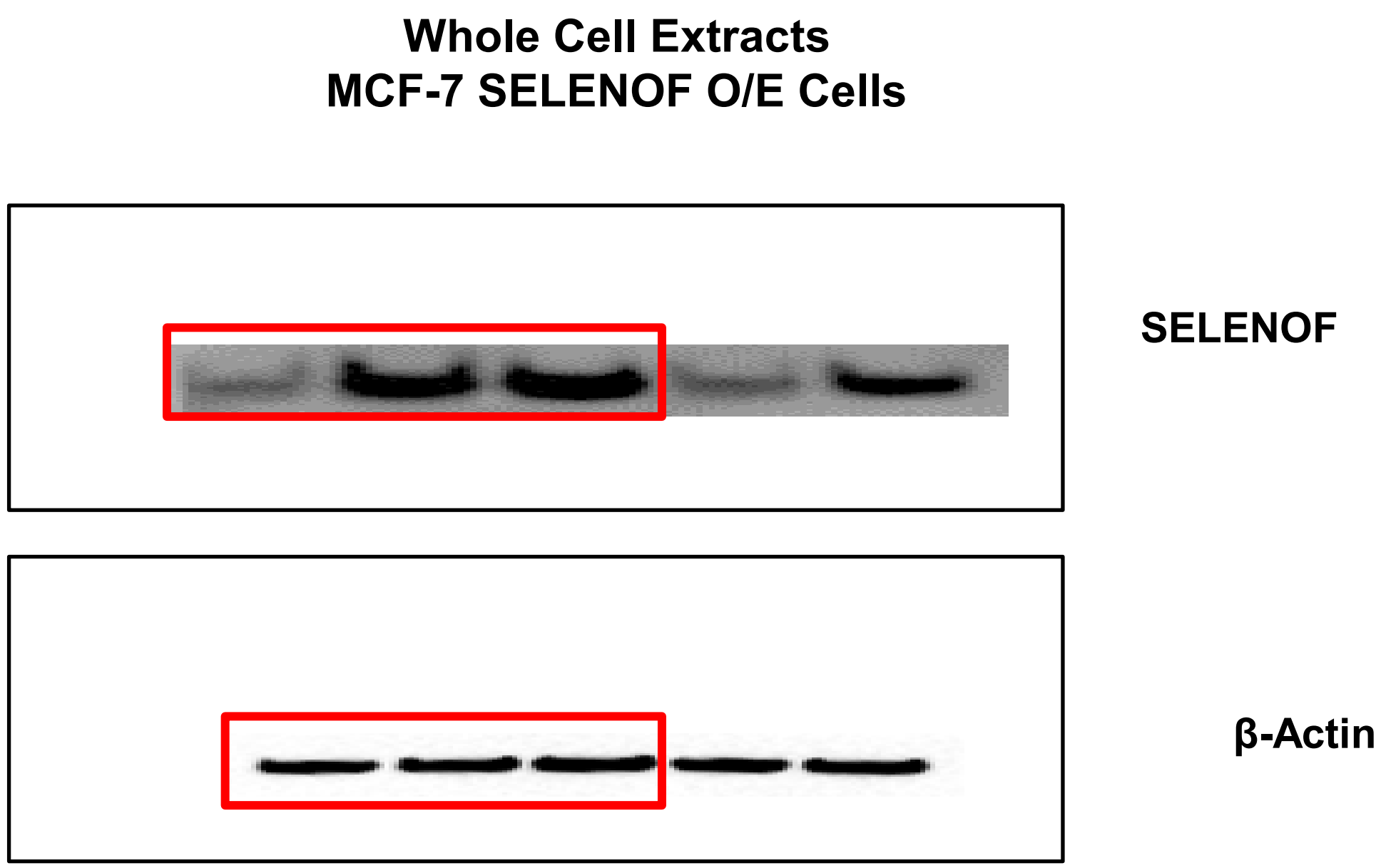

Whole Cell Extracts
MCF-7 SELENOF O/E Cells
SELENOF
β-Actin

# Supplementary Figure 1

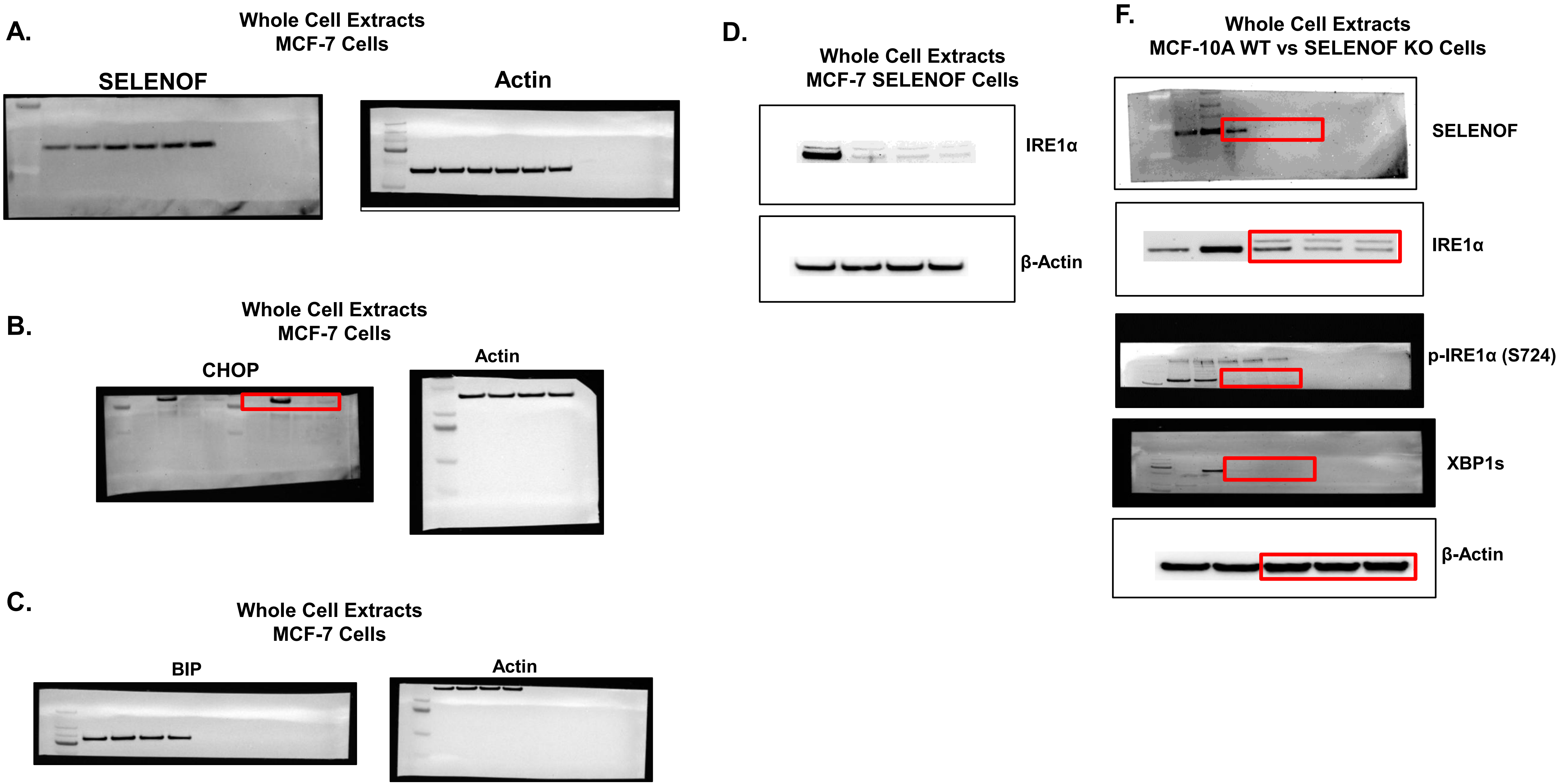

Figure 2.

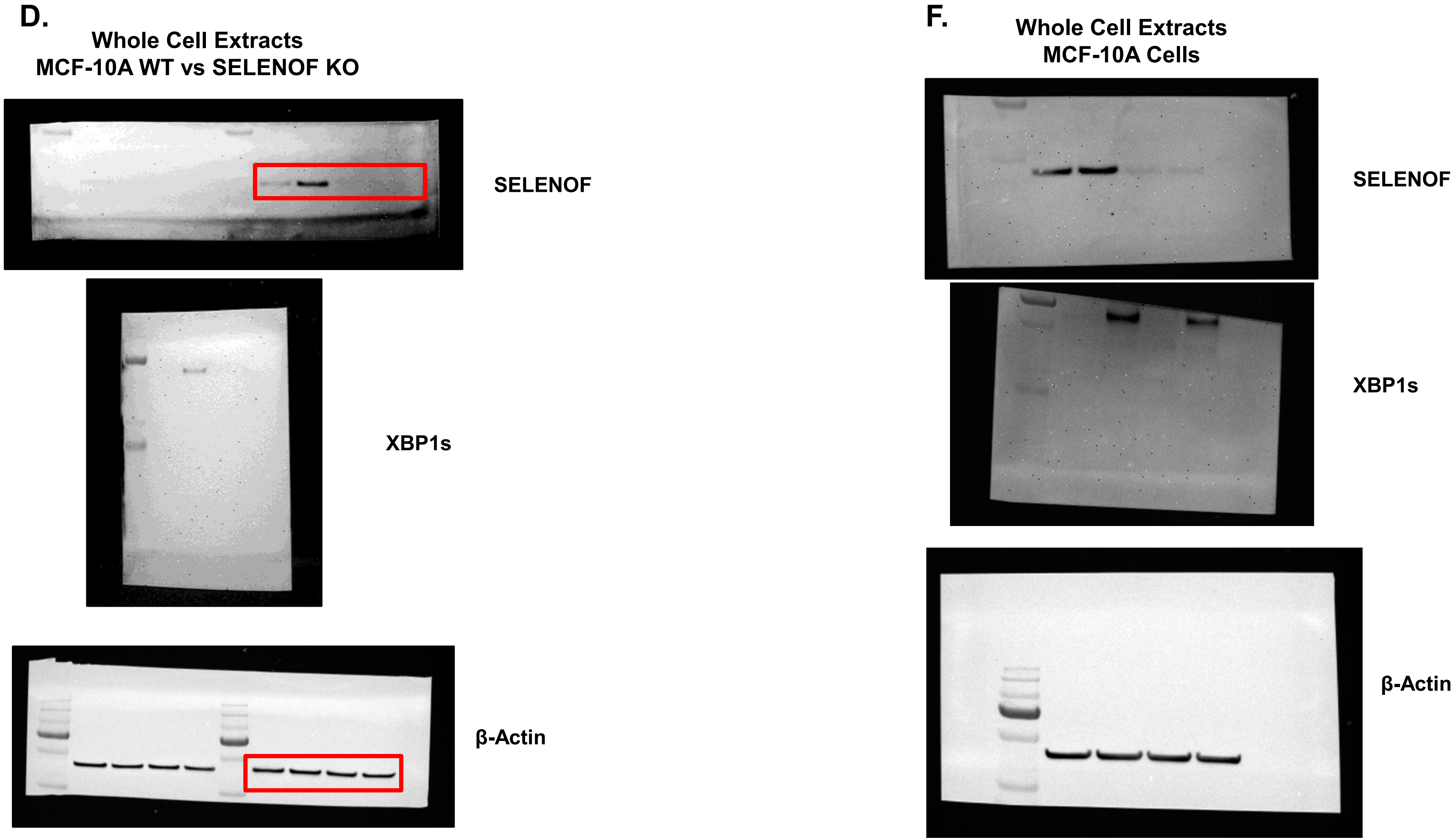
D.
Whole Cell Extracts
MCF-10A WT vs SELENOF KO
SELENOF
XBP1s
β-Actin
F.
Whole Cell Extracts
MCF-10A Cells
SELENOF
XBP1s
β-Actin

Supplementary Figure 2

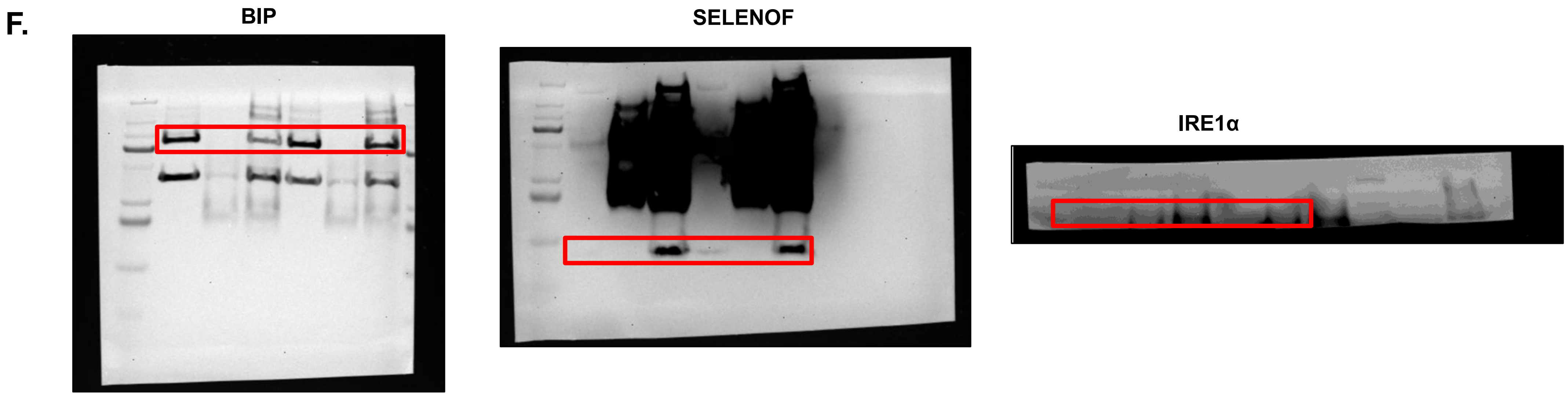

Figure 3.

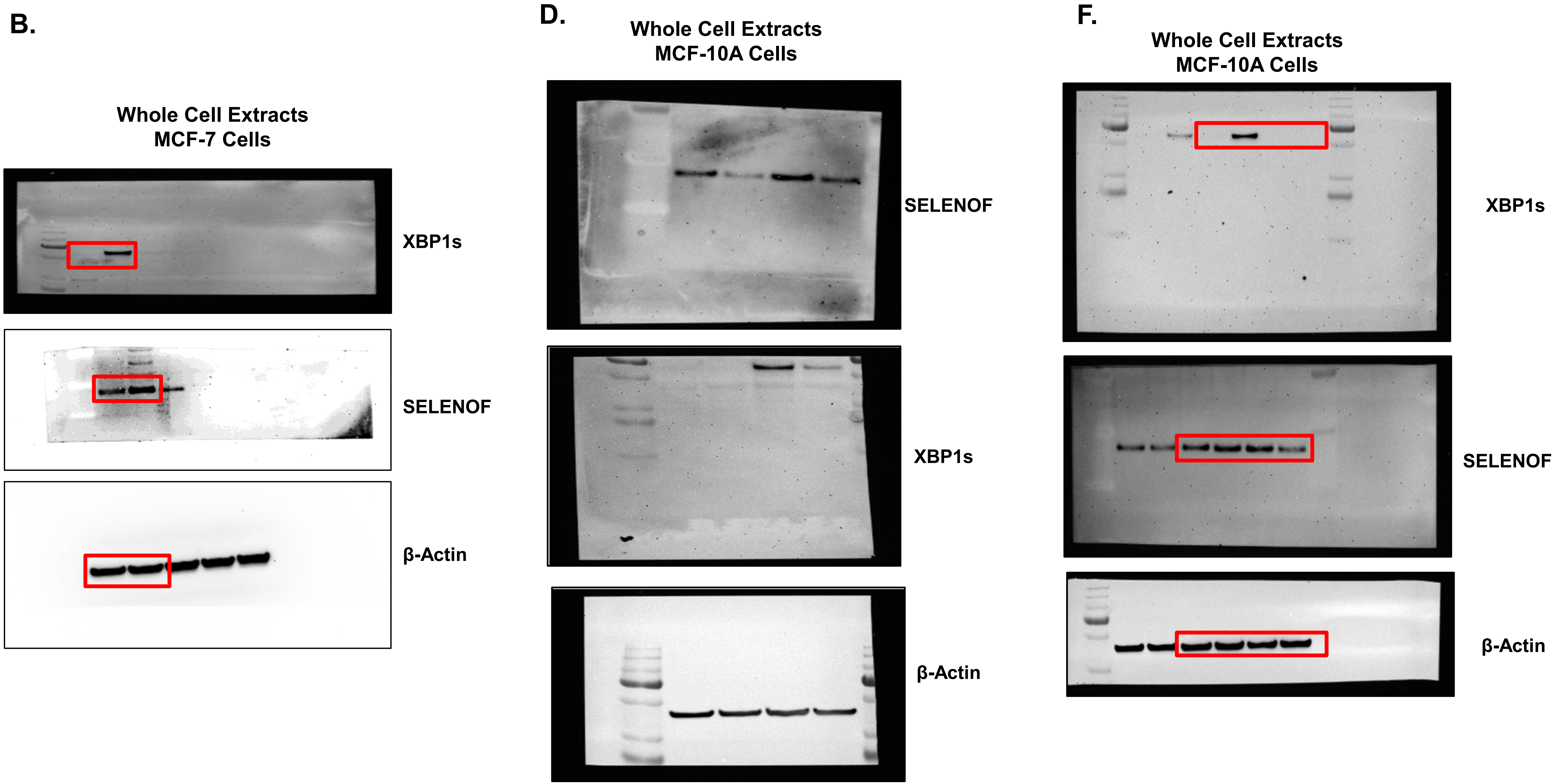

B.
Whole Cell Extracts
MCF-7 Cells
XBP1s
SELENOF
β-Actin
D.
Whole Cell Extracts
MCF-10A Cells
SELENOF
XBP1s
β-Actin
F.
Whole Cell Extracts
MCF-10A Cells
XBP1s
SELENOF
β-Actin

Figure 5.

**D.**

**Whole Cell Extracts**
**MCF-10A Cells**

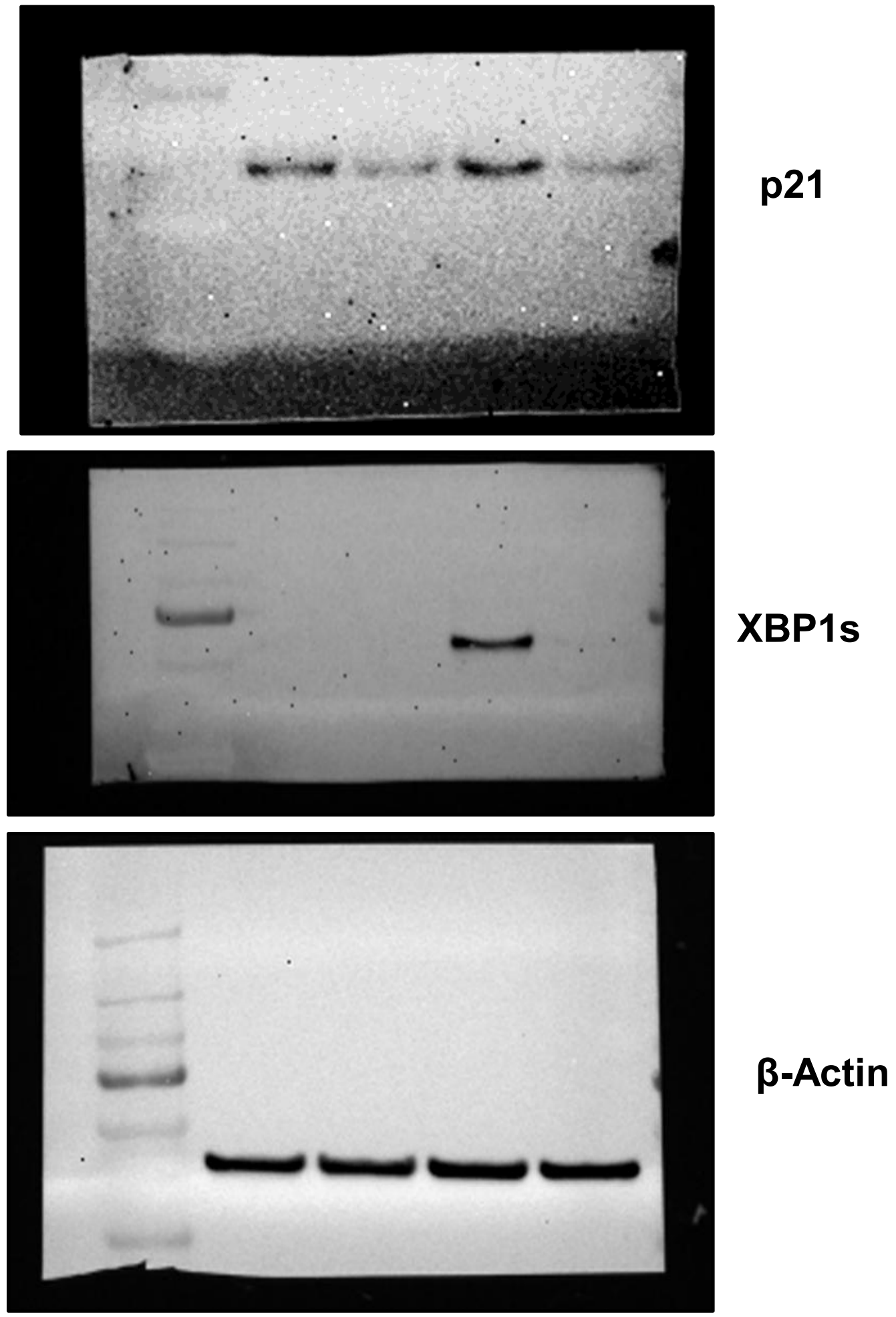


Figure 6.

**A.**

**Whole Cell Extracts**
**MCF-10A Cells**

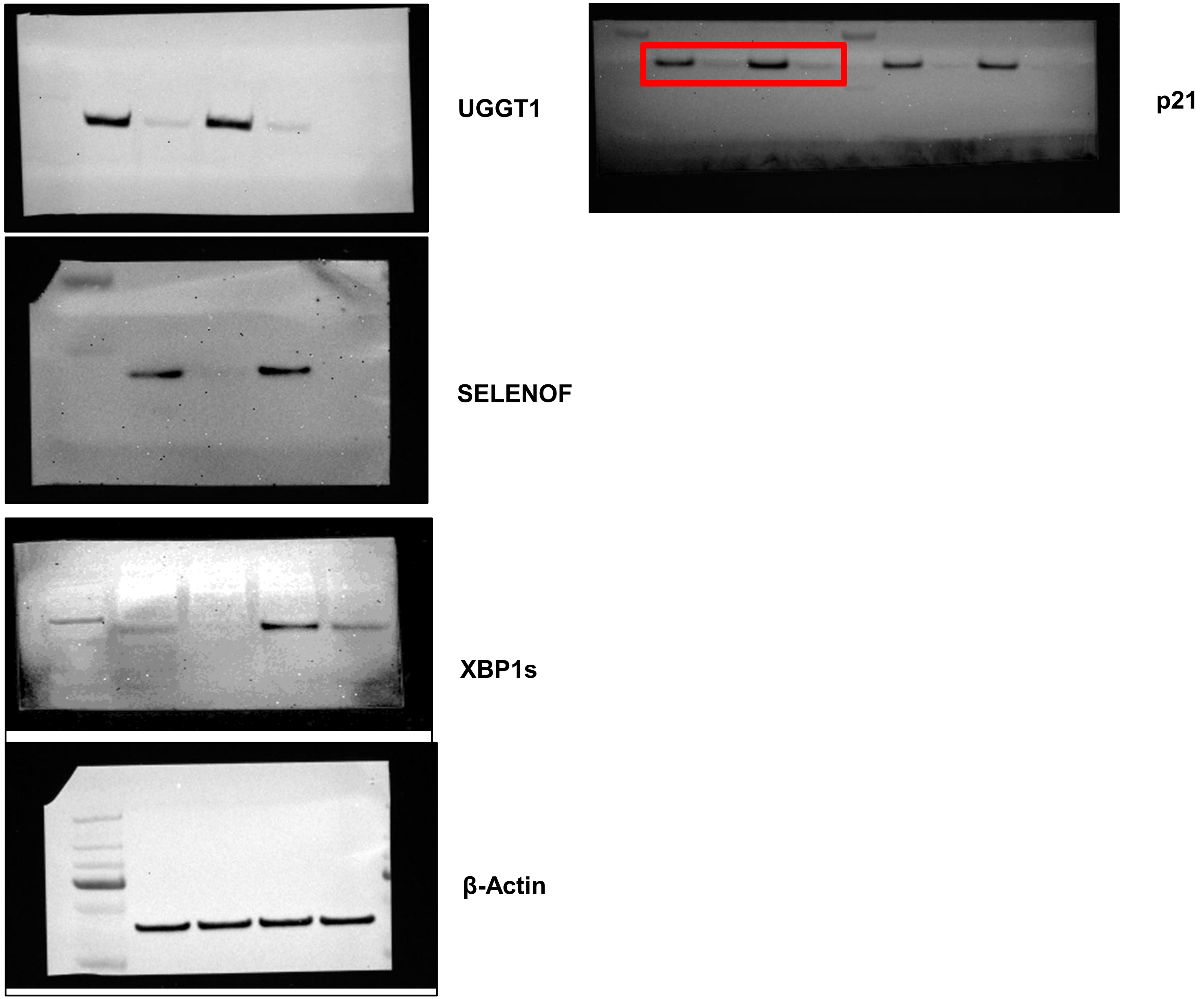

Supplement: Supplementary file 2 [file 41420_2026_3285_MOESM3_ESM.pdf]
