# Supplementary material for: Crosstalk between SELENOF and the unfolded protein response in breast epithelium dictates cell fate
Source: Cell Death Discov. 2026 Aug 10;12:445. doi: 10.1038/s41420-026-03285-7 (PMC13740312; doi:10.1038/s41420-026-03285-7)
Supplement: Supplementary file 3 [file 41420_2026_3285_MOESM1_ESM.docx]

**Supplementary Figure 1.** (A) SELENOF levels in MCF-7 cells treated with 50 nM sodium selenite for the indicated time points were determined by western blotting. Protein densitometry values are shown below the bands. β-Actin is a loading control. (B-C) CHOP and BIP protein levels were determined in MCF-7 cells treated with 1µM thapsigargin in the presence of either a PERK inhibitor, 1 µM GSK2606414 in (B) or an ATF6 inhibitor, 20 µM CEAPIN-A7 in (C). Protein densitometry values are shown below the bands. (D) IRE1α levels were determined in MCF-7 SELENOF cells transfected with different concentrations of siIRE1α. (E) Cells viability of MCF-10A WT and SELENOF KO cells was determined by crystal violet following treatments with IRE1α inhibitor (STF083010, 2 µM; 4μ8c, 0.1 µM), PERK inhibitor (GSK2606414, 2 µM) or ATF6 inhibitor (CEAPIN-A7, 6 µM) for 3 days. (F) SELENOF, IRE1α, p-IRE1α, and XBP1s levels were determined in MCF-10A WT and SELENOF KO cells. (G) Baseline *XBP1s* mRNA expression was determined in MCF-10A WT and SELENOF KO cells.

**Supplementary Figure 2.** (A-E) *EDEM1*, *PERK*, *ATF4*, *CHOP*, and *GADD34* mRNA expressions were determined in MCF-10A WT and SELENOF KO cells treated with or without thapsigargin at indicated concentrations for 24 hours. (F) Interaction of SELENOF with BIP or IRE1α was assessed by immunoprecipitation using SELENOF antibody–conjugated beads or IgG control beads in MCF-10A WT cells treated with or without 1 μM thapsigargin for 6 hours. Data represents the mean ± SEM of independent experiments (n=3). **P*≤0.05, ***P*≤0.01, ****P*≤0.001, *****P* ≤0.0001, NS is not significant.

**Supplementary Figure 3.** (A) *XBP1s* mRNA expression was determined in breast cancer cell lines, MDA-MB-231 and HCC1806, treated with or without thapsigargin (Thapsi, 1 μM for 24 hours). (B) *XBP1s* mRNA expression was determined in MCF-10A cells transfected with siNeg or siXBP1, 10 nM each for 24 hours, followed by treatment with or without 1 μM thapsigargin for 24 hours. (C-D) Correlations between *SELENOF* and *DNAJB9* (B) or *SELENOF* and *EDEM1* (D) mRNAs were evaluated in breast cancer patient samples within the METABRIC database, n=1904 samples. Spearman and Pearson coefficients and *P* values were indicated. Data represents the mean ± SEM of independent experiments (n=3). ****P* ≤0.001, *****P* ≤0.0001.

**Supplementary Figure 4.** (A) Genotyping gel for the Selenof WT mice and KO mice. Upper band indicates the presence of a Neo cassette, which was used to knockout Selenof. Lower band indicates the presence of Selenof. If just the Neo cassette band is present, this indicates a Selenof KO mouse. If just the Selenof band is present, this indicates a WT mouse. If both bands are present, this indicates a heterozygous mouse. (B-D) *Perk*, *Atf4* and *Gadd34* mRNA expressions were determined in mammary glands from WT or Selenof KO mice treated with or without 0.8 μg/g thapsigargin for 16 hours. (E) *PERK*, *ATF4* and *GADD34* mRNA expressions were determined in xenograft tumors established from MCF-7 with or without SELENOF overexpression. Data represents the mean ± SEM of independent experiments. **P* ≤0.05, ****P* ≤0.001, *****P* ≤0.0001.

**Supplementary Figure 5.** (A-F) *Selenof* mRNA and expression of other UPR genes were evaluated by RT-QPCR in liver tissues from WT and Selenof KO mice treated with or without 0.8 μg/g thapsigargin for 16 hours. Data represents the mean ± SEM of independent experiments. **P* ≤0.05, *****P* ≤0.0001, NS is not significant, ND is not detected.

**Supplementary Figure 6.** (A) Apoptosis was determined by flow cytometry in MCF-10A cells treated with stained with propidium iodide (PI) and annexin V. Cells were treated with varying concentrations of thapsigargin for 24 hours. (B-C) Cell gating strategies for data shown in Figure 5A and in Figure 5B are indicated.

**Supplementary Figure 7.** Senescence associated β-galactosidase positive cells were determined by X-Gal staining of MCF-10A WT and SELENOF KO cells treated with 5 nM thapsigargin for 72 hours. Representative pictures are shown on the right. Data represents the mean ± SEM of independent experiments. **P* ≤0.05, ****P* ≤0.001, *****P* ≤0.0001.
